# Dual‐Mode Nanoporous SiO_2_ Memristors with Coexisting Volatile and Nonvolatile Dynamics for Reservoir Computing

**DOI:** 10.1002/advs.76163

**Published:** 2026-06-18

**Authors:** Bohao Ding, Tongjun Zhang, Li Shao, Nikolay Zhelev, Andrew L. Hector, Ruomeng Huang

**Affiliations:** ^1^ School of Electronics and Computer Science University of Southampton Southampton UK; ^2^ School of Chemistry and Chemical Engineering University of Southampton Southampton UK

**Keywords:** memristor, nanoporous SiO_2_, neuromorphic computing, reservoir computing system

## Abstract

Energy‐efficient and adaptive neuromorphic hardware requires material platforms that can intrinsically integrate transient neural dynamics with stable long‐term memory within a single device architecture. Here, a cross‐point nanoporous SiO_2_ memristor that unifies volatile and nonvolatile switching behaviors within a single material platform is reported. The engineered nanoporous framework provides well‐defined ion migration pathways, enabling controlled modulation of conductive filaments and reversible transitions between short‐term plasticity (STP) and long‐term plasticity (LTP) through simple compliance‐current tuning. Leveraging this dual‐mode functionality, the volatile dynamics of the nanoporous SiO_2_ memristors are employed directly as a physical reservoir, while the nonvolatile conductance states serve as synaptic weights in the readout layer. Using a conductance‐aware training scheme, reservoir computing (RC) is demonstrated on the same device platform, achieving 93.7% accuracy in MNIST handwritten‐digit recognition. Beyond standard benchmark datasets, the system further enables ECG temporal biosignal classification, reaching over 88% accuracy in distinguishing normal and abnormal heartbeat patterns. These results establish a single‐material, CMOS‐compatible neuromorphic platform capable of integrating dynamic processing with persistent memory, offering a scalable and low‐power pathway toward compact intelligent edge computing hardware.

## Graphical abstract

A nanoporous SiO_2_ memristor enabling reconfigurable volatile and non‐volatile switching within a single device is demonstrated. The dual‐mode functionality supports both physical reservoir dynamics and synaptic weight storage, allowing unified hardware implementation of reservoir computing for temporal information processing, including image and biosignal classification.

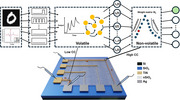

## 1 Introduction

The exponential proliferation of spatiotemporal data streams, ranging from biosignals and speech to video and distributed sensor networks, has created an urgent demand for energy‐efficient, low‐latency information processing hardware [1, 2]. Conventional von Neumann architectures, inherently constrained by the physical separation of memory and computation, suffer from excessive energy dissipation and severe data‐transfer bottlenecks during intensive read–write operations [3]. As data generation continues to outpace Moore's law scaling, these inefficiencies have catalyzed the rise of neuromorphic computing, which emulates the brain's event‐driven and in‐memory processing to achieve massive parallelism and ultralow power consumption [4, 5, 6, 7].

Among emerging neuromorphic frameworks, reservoir computing (RC) has attracted particular attention for temporal classification and prediction, especially in edge‐computing scenarios [8, 9]. Unlike conventional recurrent neural networks, RC employs a high‐dimensional nonlinear dynamical system (the reservoir) whose internal parameters remain fixed, while only a linear readout layer is trained [10]. This separation of dynamics from learning significantly reduces training complexity and enables rapid adaptation. When implemented physically, RC systems harness the intrinsic device physics of materials to project temporal inputs into rich dynamical trajectories, thereby reducing reliance on power‐intensive numerical computation [11, 12].

Memristive devices provide a natural hardware substrate for neuromorphic systems. Volatile memristors emulate short‐term plasticity (STP) through the spontaneous formation and relaxation of conductive filaments, reproducing the temporal dynamics of biological synapses that are essential for physical RC. These devices have been successfully employed as dynamic reservoirs for processing speech, biosignals, and sequential patterns [13, 14, 15, 16]. In contrast, nonvolatile memristors exhibit long‐term plasticity (LTP), offering stable and programmable conductance states that are indispensable for the readout layers in RC and other neuromorphic networks [17, 18, 19, 20]. Despite this functional complementarity, most reported memristor‐based RC systems still rely on heterogeneous device combinations or off‐chip electronics—using volatile elements for reservoir dynamics and separate nonvolatile memories for weight storage [21, 22, 23, 24, 25]. Comparatively few studies have demonstrated unified memristor platforms that simultaneously support pulse‐driven STP/STD and LTP/LTD functionalities for temporal‐information‐processing applications. As summarized in Table S1, most existing systems do not achieve the simultaneous integration of volatile reservoir dynamics and nonvolatile, trainable synaptic weight adaptation within a single device architecture, particularly for applications involving dynamic biosignal processing.

Nanoporous materials offer a promising route to achieve such unified memristive dynamics. Their interconnected pore networks offer abundant ionic diffusion pathways, large surface‐to‐volume ratios, and localized electric‐field enhancement, enabling precise control over filament nucleation, dissolution kinetics, and charge storage [26, 27, 28, 29, 30]. Although resistive switching phenomena have also been widely reported in conventional dense SiO_2_ based memristors [31, 32, 33], structural porosity provides additional tunability over filament relaxation and ionic redistribution through spatial confinement effects. Structural parameters such as pore size, orientation, and connectivity critically determine filament stability and retention characteristics, allowing a single device framework to support both STP and LTP behaviors under different operating conditions [34, 35, 36]. Previous studies have shown that nanoporous architectures in metal oxides such as TaO_x_, WO_x_, and TiO_2_ enhance switching uniformity, linear analog conductance modulation, and device endurance, validating the materials design principle of using structural porosity to tailor ionic dynamics [37, 38, 39].

In parallel, recent studies have shown that oxide‐based heterostructure memristors can improve switching uniformity and neuromorphic functionality through engineered heterointerfaces that modulate defect distribution and ion migration dynamics [40, 41]. These studies provide valuable insights into the role of interface engineering in tailoring memristive behaviors for advanced neuromorphic applications. Compared with these multilayer heterostructure systems, the nanoporous SiO_2_ platform investigated in this work offers a structurally simpler and fully CMOS‐compatible single‐material architecture while still enabling tunable dual‐mode switching dynamics within the same device framework.

Among various porous oxide systems, nanoporous silica stands out due to its facile sol–gel synthesis, fine tunability of porosity, and CMOS compatibility, offering a scalable pathway to wafer‐level neuromorphic integration [42, 43, 44]. Prior work—including ours—has shown that nanostructural engineering of SiO_2_ enables controllable tuning of switching volatility by modulating pore geometry and local bonding environment, thereby influencing filament stability and relaxation kinetics [45, 46, 47]. Together, these attributes position nanoporous silica as a promising platform for developing dual‐mode memristors that integrate transient and persistent memory behaviors within a single, scalable material platform.

In this work, we report nanoporous silica‐based dual‐mode cross‐point memristors in which volatile and nonvolatile switching behaviors can be electrically reconfigured by tuning the forming compliance current. This reconfigurability further enables neuromorphic functionalities, including volatile STP/STD responses for temporal‐information processing and nonvolatile LTP/LTD weight modulation for stable synaptic adaptation. Building on this unified device‐level functionality, we establish a neuromorphic computing framework in which the same nanoporous SiO_2_ memristor operates in STP mode as a physical reservoir for time‐multiplexed feature projection and in LTP mode as the readout layer with conductance‐based synaptic weights. The feasibility of this approach is demonstrated through two representative tasks: i) MNIST handwritten‐digit recognition, achieving a classification accuracy of 93.7%, and ii) ECG temporal biosignal classification, achieving an accuracy exceeding 88% using experimentally measured pulse‐encoded device responses. By integrating volatile reservoir dynamics and nonvolatile synaptic weight adaptation within a unified memristive architecture, this work provides a scalable route toward hardware‐oriented temporal neuromorphic computing and biosignal‐processing systems.

## 2 Results and Discussion

**FIGURE 1:**
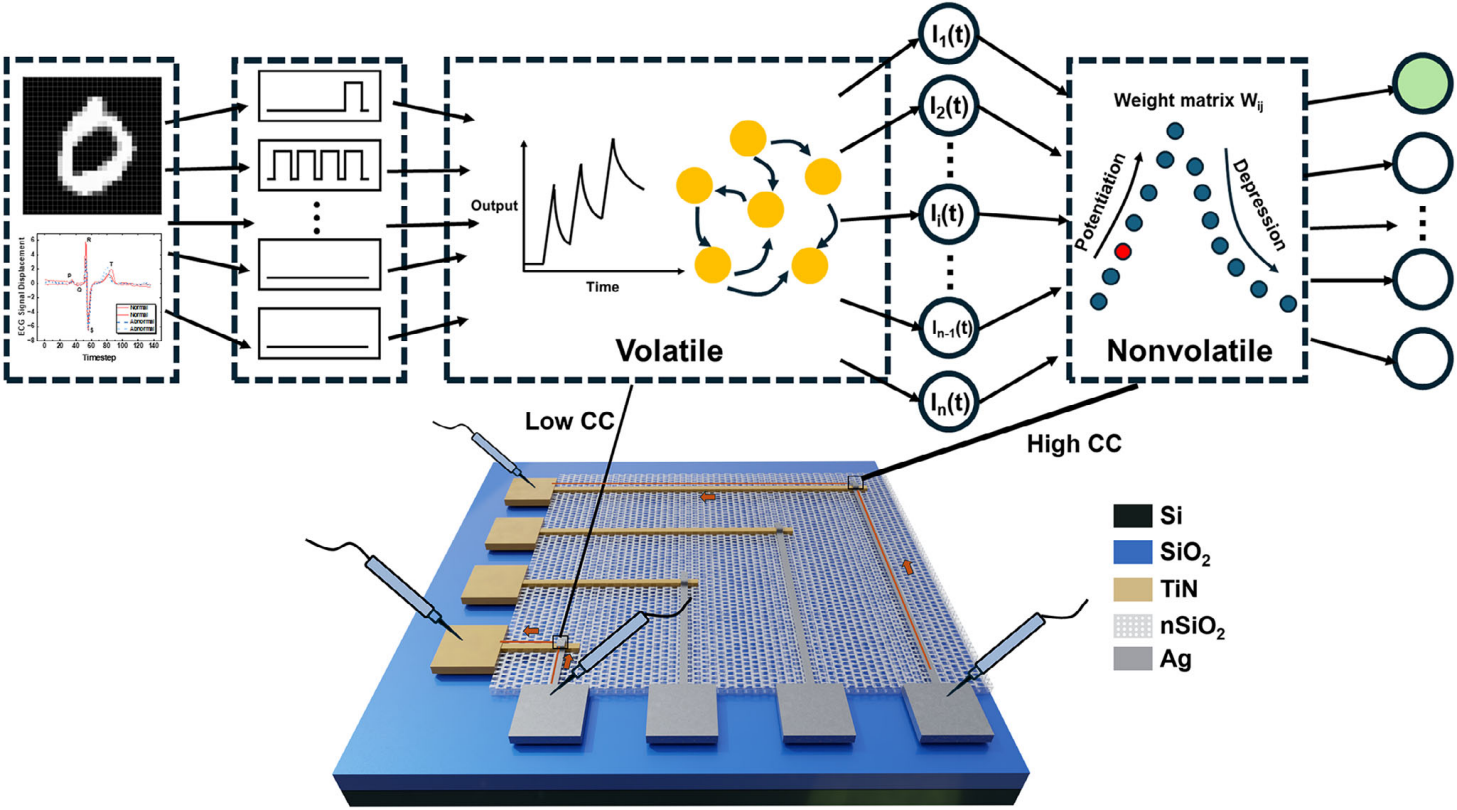
Overall architecture and workflow of the unified nanoporous SiO_2_ memristor‐based neuromorphic system for MNIST digit and ECG signal recognition.

Figure 1 presents an overview of the device‐to‐algorithm framework that links the physical properties of nanoporous SiO_2_ memristors with system‐level neuromorphic computation. In this architecture, spatiotemporal inputs including handwritten‐digits from the MNIST dataset and physiological electrocardiogram (ECG) signals are first preprocessed and time‐encoded into pulse sequences *I*(*t*). These pulse trains drive the cross‐point nSiO_2_ memristors, whose volatile dynamics serve as the physical reservoir, while their experimentally measured nonvolatile conductance states are used to implement the adaptive readout layer.

By tuning the compliance current (CC), the same device can be electrically programmed into distinct operational regimes: a low CC induces volatile, short‐term dynamic responses that emulate synaptic dynamics suitable for reservoir computing, whereas a high CC enables nonvolatile, long‐term conductance modulation that functions as programmable synaptic weights for classification. This dual‐mode behavior allows both temporal information encoding and synaptic weight representation to be performed directly in the same device architecture, without the need for heterogeneous material integration. The subsequent sections detail the device architecture, structural characterization, and neuromorphic performance of the nanoporous SiO_2_ cross‐point system.

### 2.1 Structural and Electrical Characteristics of Nanoporous SiO_2_ Memristors

**FIGURE 2:**
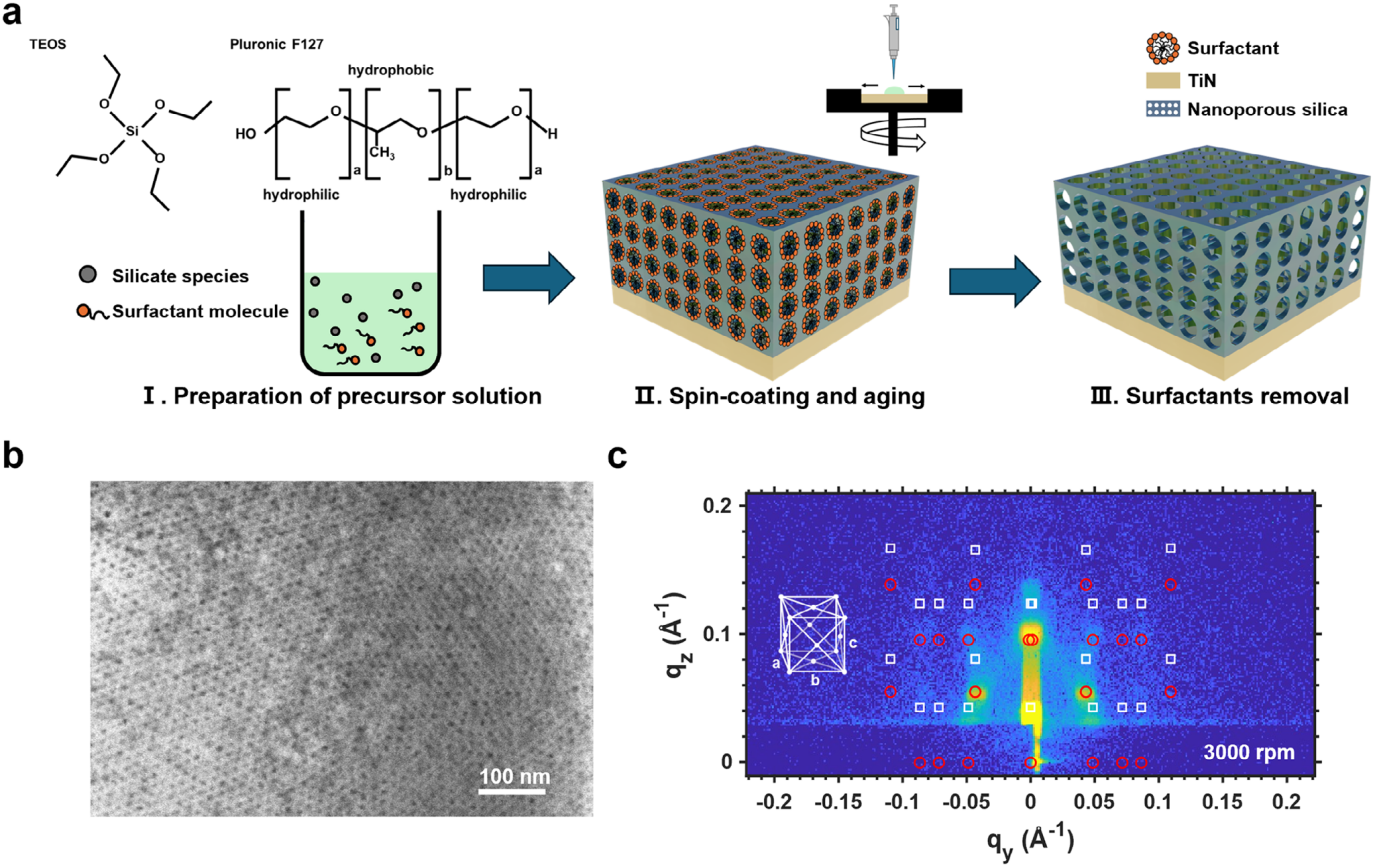
Structural characterization of the 3D‐ordered nanoporous silica (nSiO_2_) thin films. (a) Schematic illustration of the sol–gel fabrication process of nanoporous SiO_2_ thin films. (b) Top‐view SEM image showing the uniform nanoporous architecture. (c) GISAXS pattern of the SiO_2_ thin film with simulated Bragg reflections confirming the periodic nanostructure.

Three‐dimensional nanoporous SiO_2_ thin films were synthesized via the Stöber sol‐gel process [48], using tetraethyl orthosilicate (TEOS) as the silica precursor and Pluronic F‐127 functioning as both the structure‐directing surfactant and template, as shown in Figure 2a [15, 45]. The precursor solution was spin‐coated onto patterned TiN/SiO_2_/Si substrates, and the detailed processing conditions are provided in the *Experimental Section*. High‐resolution scanning electron microscopy (SEM) (Figure 2b) reveals a uniform film surface decorated with nanopores of approximately 8 nm in diameter, confirming successful pore formation and conformal coverage on the substrate. The grazing‐incidence small‐angle X‐ray scattering (GISAXS) pattern (Figure 2c) exhibits sharp diffraction maxima corresponding to transmitted (red circles) and reflected (white squares) Bragg peaks, indicative of a highly ordered three‐dimensional pore network. Indexing of these peaks identifies an orthorhombic *Fmmm* symmetry with lattice parameters a = 176 Å, b = 147 Å, and c = 260 Å, and the majority of domains are oriented with the [010] axis normal to the substrate. This structural ordering confirms the preservation of a well‐defined three‐dimensional nanoporous architecture throughout the film.

X‐ray photoelectron spectroscopy (XPS) of Si 2p and O 1s (Figure S1) further confirms the chemical composition. The Si 2p spectrum (Figure S1a) shows a single dominant peak centered at ≈103.5 eV, characteristic of fully oxidized Si–O bonding in SiO_2_. In the O 1s region (Figure S1b), two distinct components corresponding to O–Si (532.9 eV) and O–H (534.0 eV) are observed, indicating the coexistence of framework oxygen species and hydroxyl‐related surface states. The presence of –OH groups has been reported to play an important role in filamentary switching of nanoporous SiO_2_‐based memristors, where surface hydroxyls and adsorbed moisture can regulate ion migration pathways and local defect distribution. In particular, –OH groups can act as charge‐trapping sites, altering the local electric field distribution and consequently influencing filament dynamics and switching stability [49, 50].

Ellipsometry measurements show that spin‐coating at 3000 rpm yields a film thickness of ca. 120 nm, obtained by fitting the data with the Cauchy dispersion model (Figure S2a,b). Compared with thermally grown SiO_2_ (*n* = 1.43), the nanoporous film exhibits a reduced refractive index of 1.28 at 800 nm, reflecting the presence of low‐refractive‐index components such as air and adsorbed moisture within the pore network (Figure S2c). The porosity was estimated using the Lorentz–Lorenz effective medium approximation, in which the film is treated as an optically homogeneous mixture of SiO_2_ and voids. Under the assumption that the pore size is much smaller than the wavelength of light, the porosity can be expressed as:

(1) $$Porosity=1-\frac{n_{i}^{2}-1}{n_{d}^{2}-1}$$

where *n_i_* is the refractive index of the porous silica film, and *n_d_* is the refractive index of the thermally grown SiO_2_ layer [51]. The calculated porosity is ≈38%. Collectively, these results demonstrate that uniform, well‐ordered nanoporous SiO_2_ films can be reliably deposited on patterned substrates via a simple 3000 rpm spin‐coating process.

**FIGURE 3:**
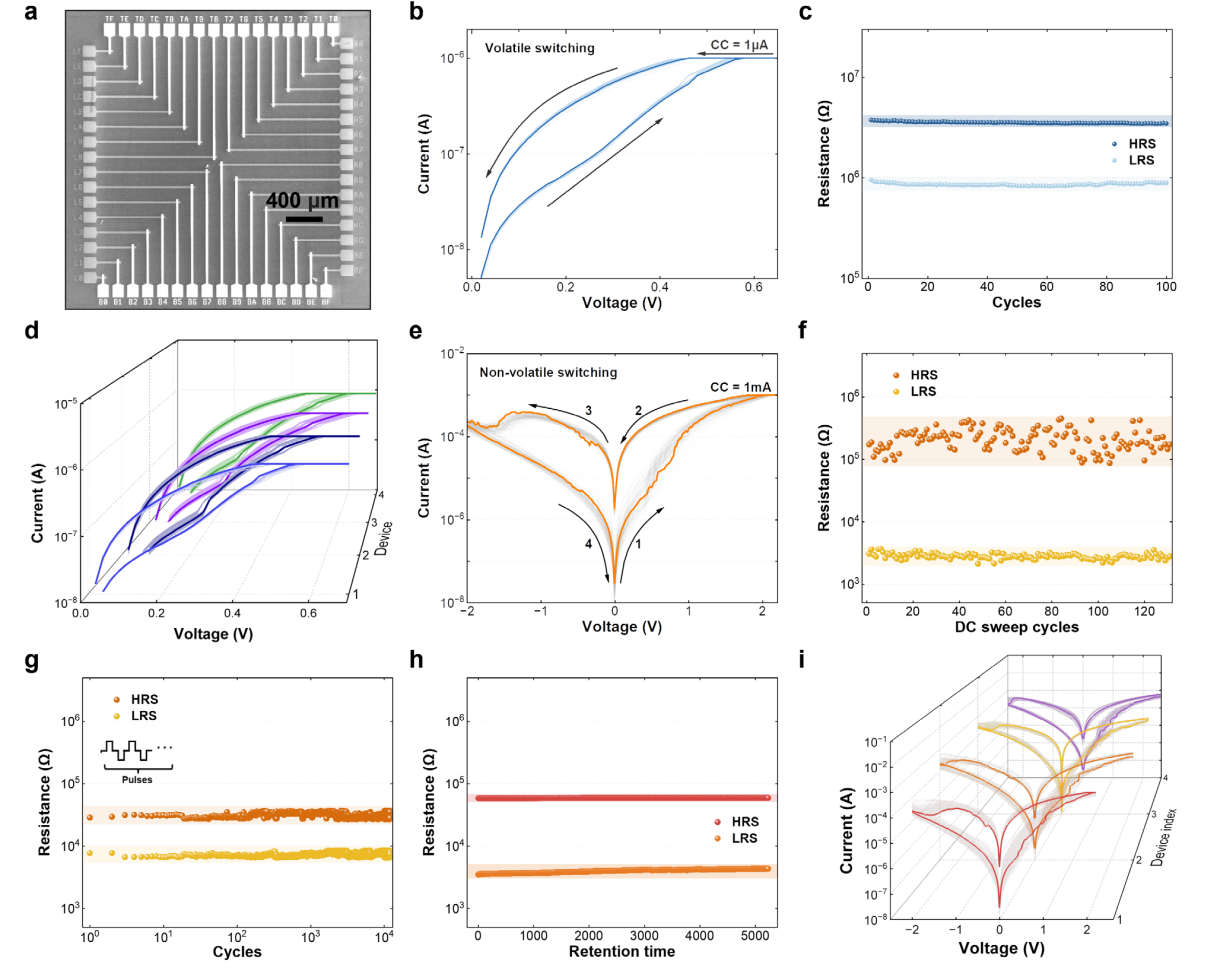
Resistive switching characteristics of the nanoporous SiO_2_ memristor. (a) Top‐view SEM image of the nanoporous SiO_2_ cross‐point device (20 µm × 20 µm). (b) Forming‐free volatile *I–V* characteristics from consecutive sweeps under a compliance current (CC) of 1 µA. (c) Endurance characteristics of the volatile mode at a 0.1 V read voltage. (d) Device‐to‐device volatile *I–V* characteristics. (e) Consecutive nonvolatile bipolar *I–V* sweeps under a CC of 1 mA. (f) Endurance characteristics of the nonvolatile switching mode under repeated DC sweep conditions measured at a read voltage of 0.1 V. (g) Endurance characteristics of the nonvolatile switching mode under pulse‐driven operation conditions. The pulse endurance measurements were performed using alternating ±3 V pulses with pulse width and interval time of 1 ms, while the resistance states were read at 0.1 V. (h) Retention characteristics of the nonvolatile mode measured under a CC of 1 mA. (i) Device‐to‐device nonvolatile *I–V* characteristics.

To implement the proposed pipeline in hardware, memristor devices were fabricated in cross‐point memristors (20 µm × 20 µm), as shown in Figure 3a (fabrication process is schematically illustrated in Figure S3 and detailed in the *Experimental Section*). The electrical switching characteristics were examined by applying DC voltage sweeps between the Ag top and TiN bottom electrodes of the nanoporous SiO_2_‐based memristors. Figure 3b presents the current–voltage (*I–V*) characteristics of a representative device operating without an electroforming step under a compliance current (CC) of 1 µA. During the forward voltage sweep from 0 to +0.7 V, the current increases sharply as the device switches from a high‐resistance state (HRS) to a low‐resistance state (LRS), followed by a spontaneous return to the HRS as the bias is reduced back to 0 V. This reversible behavior confirms volatile switching, characteristic of diffusive filament dynamics. The device exhibited stable and reproducible performance over 100 DC cycles, with the HRS and LRS measured at a read voltage of 0.1 V, maintaining a consistent resistance ratio of *≈*4 (Figure 3c). Cumulative probability analysis of the resistance states (Figure S4) reveals tightly clustered HRS and LRS distributions with moderate σ/µ values (2.72% for the LRS and 1.97% for the HRS, respectively), where µ and σ denote the mean and standard deviation of each resistance state, respectively. Device‐to‐device measurements further demonstrate reproducible volatile switching behavior across multiple devices over repeated operating cycles, with comparable switching voltages and relaxation characteristics (Figure 3d). To further evaluate endurance, extended measurements were performed over 300 consecutive cycles (Figure S5), showing reproducible switching behavior with increased cycle‐to‐cycle variation, which is characteristic of the stochastic formation and spontaneous dissolution of conductive filaments in diffusive switching systems. Despite this variation, the switching window and nonlinear dynamic response remain consistent. Additional statistical analyses of the volatile switching characteristics, including multi‐cycle *I–V* characteristics and resistance distributions across multiple devices, are provided in Figures S6 and S7, confirming consistent switching functionality under volatile operation.

When the CC was increased to 1 mA, the same device transitioned into a nonvolatile bipolar switching mode (Figure 3e). Under a positive sweep up to +2 V, the resistance gradually decreased from HRS to LRS and remained stable after the bias was removed; a reverse sweep from 0 to −2 V was required to reset the device. Figure 3f presents the DC endurance over 100 cycles. The endurance performance of the nonvolatile mode was further evaluated under pulse‐driven operation. As shown in Figure 3g, the device maintained stable resistive switching over 10^4^ pulse cycles under alternating ±3 V pulses with pulse duration and interval times of 1 ms. The resistance states, read at 0.1 V, remain clearly distinguishable throughout the measurement, indicating robust endurance performance. Retention measurements conducted for more than 5000 s at 0.1 V (Figure 3h) further confirmed the stability of the conductance states, with the HRS and LRS remaining clearly distinguishable throughout the entire measurement duration. The distributions of the HRS and LRS for DC sweep endurance, pulse endurance measurements, and retention are summarized in Figure S8, where relatively small σ/µ values further confirm the stability and reproducibility of the resistance states during nonvolatile operation. Device‐to‐device measurements (Figure 3i) further show consistent bipolar switching characteristics across multiple devices measured under a CC of 1 mA, with comparable set/reset behavior, clearly distinguishable resistance states, and a well‐defined memory window (>10). Additional statistical analyses of the nonvolatile switching characteristics are provided in Figures S9 and S10, demonstrating acceptable device‐to‐device variation. Collectively, these results demonstrate that the nanoporous SiO_2_ memristor can be reliably switched between volatile and nonvolatile operating modes through simple control of the current compliance.

The mechanism underlying the dual‐mode operation is schematically illustrated in Figure S11 and originates from current‐compliance‐controlled Ag filament formation and stability, with the nanoporous SiO_2_ framework modulating ionic transport and filament dynamics. TEM and EDX evidence from our previous work supports that the interconnected nanoporous network provides effective ion transport pathways and preferential sites for filament nucleation, facilitating Ag^+^ migration and lowering the energy barrier for filament formation [15]. At low CC, limited Ag^+^ migration leads to thin and unstable filaments that dissolve after bias removal due to ionic back‐diffusion, resulting in volatile switching. In contrast, higher CC promotes the formation of thicker and more stable filaments that persist after bias removal and require reverse bias for rupture, corresponding to nonvolatile behavior. It is worth noting that the transition between these two operating regimes is not expected to occur as an abrupt threshold process, but rather as a gradual evolution of filament stability with increasing compliance current. At intermediate CC conditions, partial filament stabilization may occur, giving rise to mixed switching characteristics with extended relaxation times or partially retained conductance states. This evolution is consistent with the electrochemical metallization (ECM) mechanism in Ag‐based memristors [52, 53]. The filamentary mechanism is further supported by transport analysis. As shown in the log–log *I–V* characteristics of our nonvolatile switching mode (Figure S12), the HRS exhibits a transition from Ohmic conduction (*I*∝*V*) to trap‐controlled space‐charge‐limited current (SCLC) behavior ($I\propto V^{2}$), followed by a trap‐filled conduction regime at higher bias, indicating that charge transport in the HRS is dominated by trap‐mediated conduction within the nanoporous SiO_2_ matrix [54, 55]. These trap states originate from intrinsic defects and are further modulated by Ag^+^ migration and local electric field redistribution. After SET, the device enters a LRS with linear *I–V* characteristics, consistent with filamentary Ohmic conduction. The coexistence of SCLC and filamentary conduction reflects the interplay between matrix‐limited transport and conductive filament pathways, a behavior widely observed in oxide‐based memristive systems, where ion migration, redox reactions, and defect‐modulated transport jointly govern filament evolution and switching behavior [56]. This structure–transport relationship establishes a coherent physical framework for understanding the dual‐mode switching characteristics of the nanoporous SiO_2_ memristor.

### 2.2 Reconfigurable Synaptic Plasticity Emulation

**FIGURE 4:**
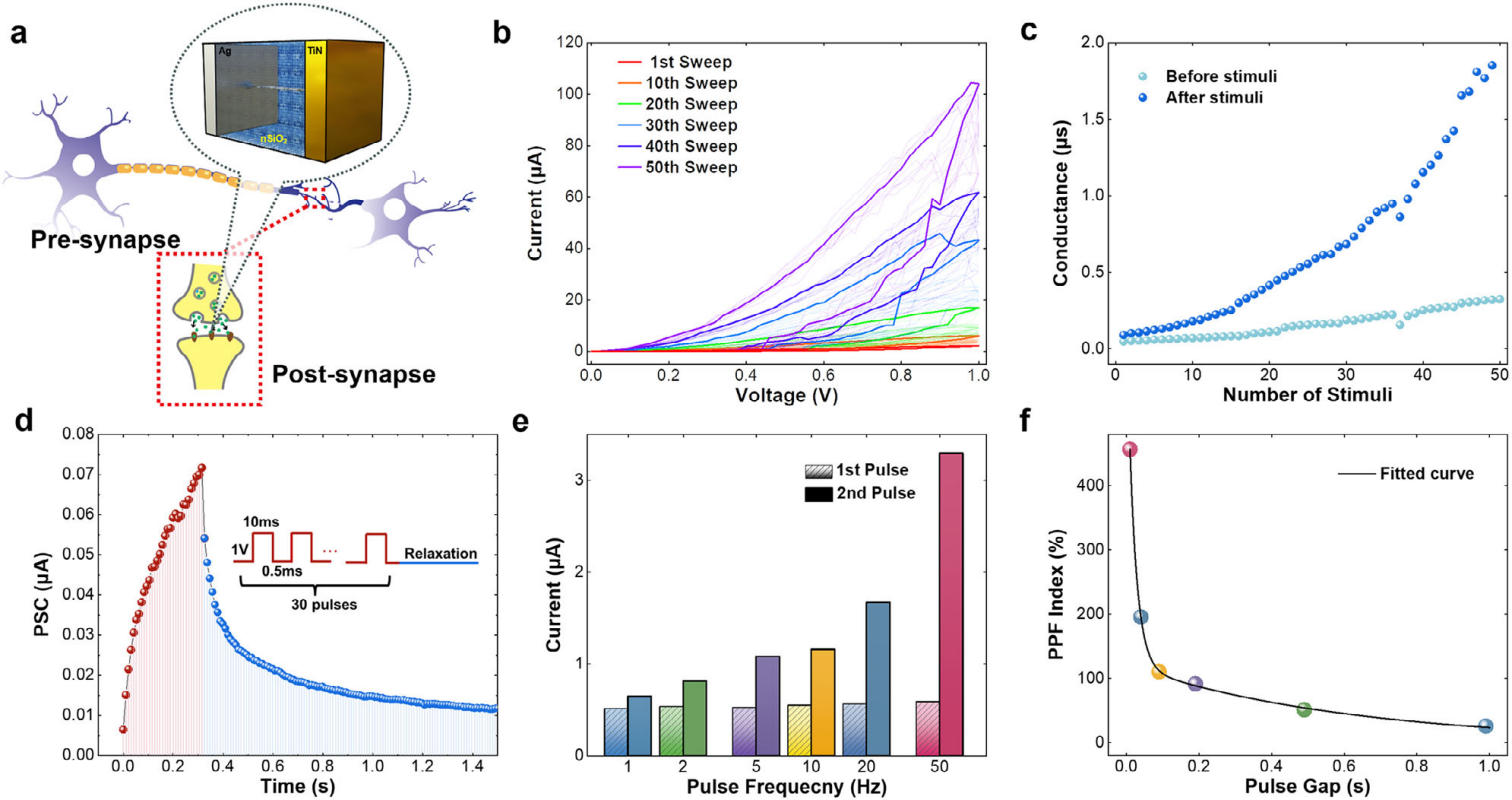
Short‐term synaptic plasticity (STP) emulated by the nanoporous SiO_2_ memristor. (a) Schematic comparison between a biological synapse and the memristor, illustrating their analogous signal transmission and modulation mechanisms. (b) Progressive I–V evolution during 50 consecutive low‐bias sweeps (0–1 V) without CC. (c) Corresponding gradual conductance potentiation extracted from panel (b), confirming analog‐like conductance modulation. (d) Incremental increase of postsynaptic current (PSC) during 30 consecutive pulse stimulations (1 V amplitude, 10 ms width, 0.5 ms interval), followed by spontaneous relaxation after pulse cessation. (e) Frequency‐dependent paired‐pulse facilitation (PPF) responses recorded under paired stimuli (1 V, 10 ms) at 1, 2, 5, 10, 20, and 50 Hz. (f) Extracted PPF index as a function of inter‐pulse interval (Δ*t*), showing a double‐exponential decay that characterizes the temporal dynamics of synaptic facilitation.

In biological neural systems, the communication between pre‐ and post‐synaptic neurons (schematically illustrated in Figure 4a) is governed by the release of neurotransmitters triggered by calcium‐ion (Ca^2+^) influx into the pre‐synaptic terminal. Upon the arrival of an action potential, voltage‐gated calcium channels open, allowing Ca^2+^ ions to enter and promote vesicle fusion at the synaptic cleft. The released neurotransmitters bind to receptors on the post‐synaptic membrane, converting the electrical signal into a chemical one and modulating synaptic conductance [57]. A defining characteristic of this process is STP, which refers to transient and activity‐dependent changes in synaptic strength that enable neural circuits to encode temporal correlations and dynamically adapt to recent stimuli. STP can manifest as facilitation, arising from residual Ca^2+^ accumulation that enhances neurotransmitter release, or as depression, caused by vesicle depletion or receptor desensitization [58]. These rapid and reversible modulations, occurring on millisecond‐to‐second timescales, form the physical substrate of biological information filtering, working memory, and adaptive computation. As illustrated in the inset of Figure 4a, analogous transient conductance dynamics can be achieved in an Ag/nSiO_2_/TiN memristive device through stimulus‐induced Ag^+^ ion migration and relaxation, providing a solid‐state counterpart to biological STP.

To emulate this function in hardware, the nanoporous SiO_2_ memristor was initially operated in its volatile mode. When a slightly higher voltage of 1.0 V was applied without CC, a series of 50 consecutive small DC sweeps (0–1 V) resulted in progressive increases in current (Figure 4b), quantified in Figure 4c as gradual conductance potentiation across successive sweeps. These results confirm that analog‐like conductance modulation can be achieved in the nSiO_2_ memristor under weak and repetitive electrical stimuli. Building on this DC‐based characterization, dynamic synaptic responses were further investigated using pulsed electrical stimulation.

As shown in Figure 4d, 30 identical voltage pulses (1 V amplitude, 10 ms duration, 0.5 ms gap) were applied to the device. The output current increased progressively with each pulse and relaxed spontaneously once the stimuli ceased, reproducing the characteristic potentiation and decay dynamics of short‐term synaptic responses. The energy consumption for each short‐term synaptic event was estimated to be ca. 0.5 nJ per pulse, indicating low‐energy operation in the volatile regime. The paired‐pulse facilitation (PPF) behavior—an essential fingerprint of STP—was further investigated by applying pairs of identical pulses (1 V, 10 ms) with varying inter‐pulse intervals (*Δt* = *1/f*). As shown in Figure 4e, the second pulse elicited a stronger current response as the stimulation frequency increased from 1 to 50 Hz, indicating frequency‐dependent facilitation (details in Figure S13). The facilitation enhancement is defined as:

(2) $$PPF\;index\;\left(\%\right)=\left(I_{2}-I_{1}\right)\;/I_{1}\times 100\%$$

where *I*_1_ and *I*_2_ represent the first and second current responses, respectively, and quantify the transient enhancement in synaptic strength. The dependence of the PPF enhancement on the inter‐pulse interval Δ*t* is well described by a double‐exponential decay function [59]:

(3) $$PPF=A_{1}\;e^{\left(\frac{-\Delta t}{\tau_{1}}\right)}+A_{2}e^{\left(\frac{-\Delta t}{\tau_{2}}\right)}$$

where τ_1_ and τ_2_ represent the characteristic relaxation times of the slow and fast decay components, respectively, and A_1_ and A_2_ are their corresponding amplitudes. The fitted parameters (A_1_ = 121.08, A_2_ = 541.44, τ_1_ = 0.611 s, τ_2_ = 0.0212 s) show good agreement with the experimental data (Figure 4f), indicating the coexistence of two distinct relaxation processes with clearly separated timescales. The fast relaxation process (τ_2_ ≈ 0.0212 s) can be attributed to rapid ionic redistribution within or around the conductive filament, giving rise to a transient residual conductive state. In contrast, the slow relaxation component (τ_1_ ≈ 0.611 s) is associated with longer‐range ionic diffusion and gradual relaxation or dissolution of the filament structure, as commonly observed in filamentary memristors governed by ion migration dynamics [60, 61].

Notably, the facilitation reaches over 400% at 50 Hz (*Δt* = 10 ms) and decreases to ≈ 25% at 2 Hz (*Δt* = 990 ms), demonstrating the device's ability to encode temporal correlations through paired‐spike timing. These results demonstrate that the memristor reproduces the essential features of biological STP, enabling dynamic and time‐dependent information processing, a foundational requirement for implementing physical reservoir computing in hardware.

**FIGURE 5:**
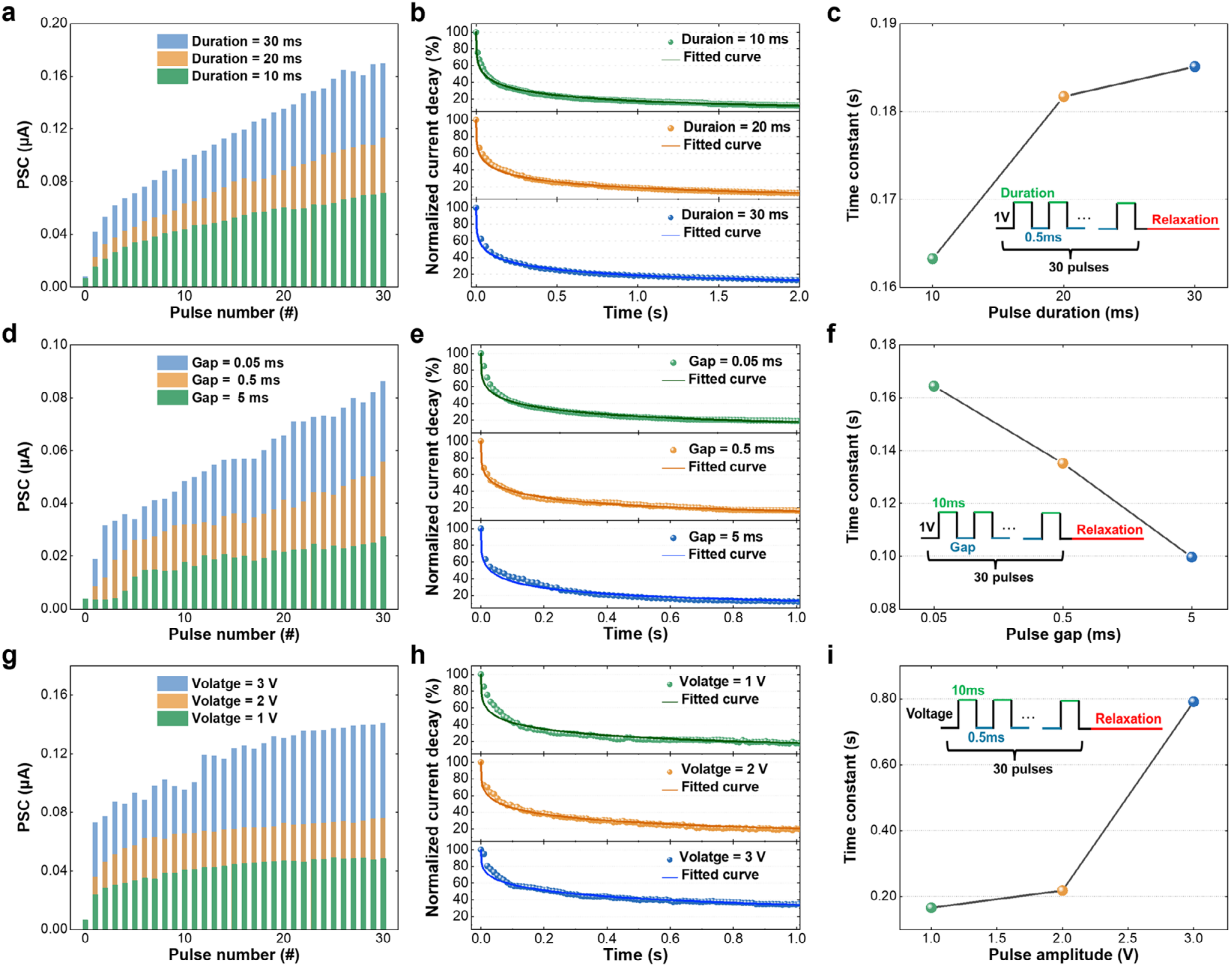
Stimulus‐dependent short‐term synaptic plasticity (STP) characteristics of the nanoporous SiO_2_ memristor. (a) PSC responses to 30 consecutive voltage pulses (1 V amplitude, 0.5 ms gap) with pulse durations varied from 10 to 30 ms, showing larger conductance potentiation for longer pulses. (b) Experimental relaxation curves fitted using a stretched‐exponential decay model, exhibiting excellent agreement. (c) Extracted relaxation time constants (τ) as a function of pulse duration. (d–f) Effect of inter‐pulse gap (0.05, 0.5, and 5 ms) on PSC amplitude and relaxation dynamics, where longer gaps allow more ion diffusion and faster recovery. (g–i) Dependence of PSC response on pulse amplitude (1–3 V), demonstrating enhanced Ag‐ion injection and prolonged decay with stronger electric fields.

Beyond demonstrating transient conductance modulation, the STP functionality in the nanoporous SiO_2_ memristor can be further tuned by varying the temporal and electrical characteristics of the input stimuli. As shown in Figure 5a, a train of 30 voltage pulses with fixed amplitude (1 V) and gap (0.5 ms) was applied while the pulse duration varied from 10 to 30 ms. Each pulse induced a gradual conductance increase, corresponding to the cumulative formation of conductive filaments, while longer pulse widths produced higher PSC peaks owing to more extensive Ag migration. After the final pulse, the current relaxed spontaneously, reflecting the self‐decay of unstable filaments. The relaxation dynamics can be fitted using a stretched‐exponential decay function [59, 62]:

(4) $$I^{*}=\frac{I}{I_{0}}=e^{-{\left(\frac{t}{\tau }\right)}^{\beta }}$$

where I* is the normalized current at time *t*, *I*_0_ is the initial current immediately after stimulation, *I* is the relaxation current at a given time *t*, τ is the relaxation time constant, and the stretching exponent β (0 < β < 1) characterizes the distribution of relaxation processes determined by the microscopic energy landscape of the material.

The fitted curves (Figure 5b) exhibit great agreement with experimental data using a constant β = 0.3, demonstrating the reliability of the model. As the pulse duration increased, the extracted relaxation constant τ also increased (Figure 5c), indicating slower self‐recovery dynamics due to more persistent filament structures. The influence of inter‐pulse gap and pulse amplitude is shown in Figure 5d–i. Larger temporal gaps (0.05, 0.5, and 5 ms) promoted greater back‐diffusion of Ag^+^ ions between stimuli, resulting in reduced PSC amplitudes and faster relaxation. Conversely, increasing the pulse amplitude from 1 to 3 V strengthened the electric‐field‐driven injection of Ag ions into the nanoporous network, leading to larger PSCs and prolonged decay times. These observations collectively indicate that the strength and duration of short‐term synaptic responses in the nanoporous SiO_2_ memristor can be precisely modulated by pulse width, amplitude, and timing, mirroring the dynamic, stimulus‐dependent plasticity of biological synapses.

**FIGURE 6:**
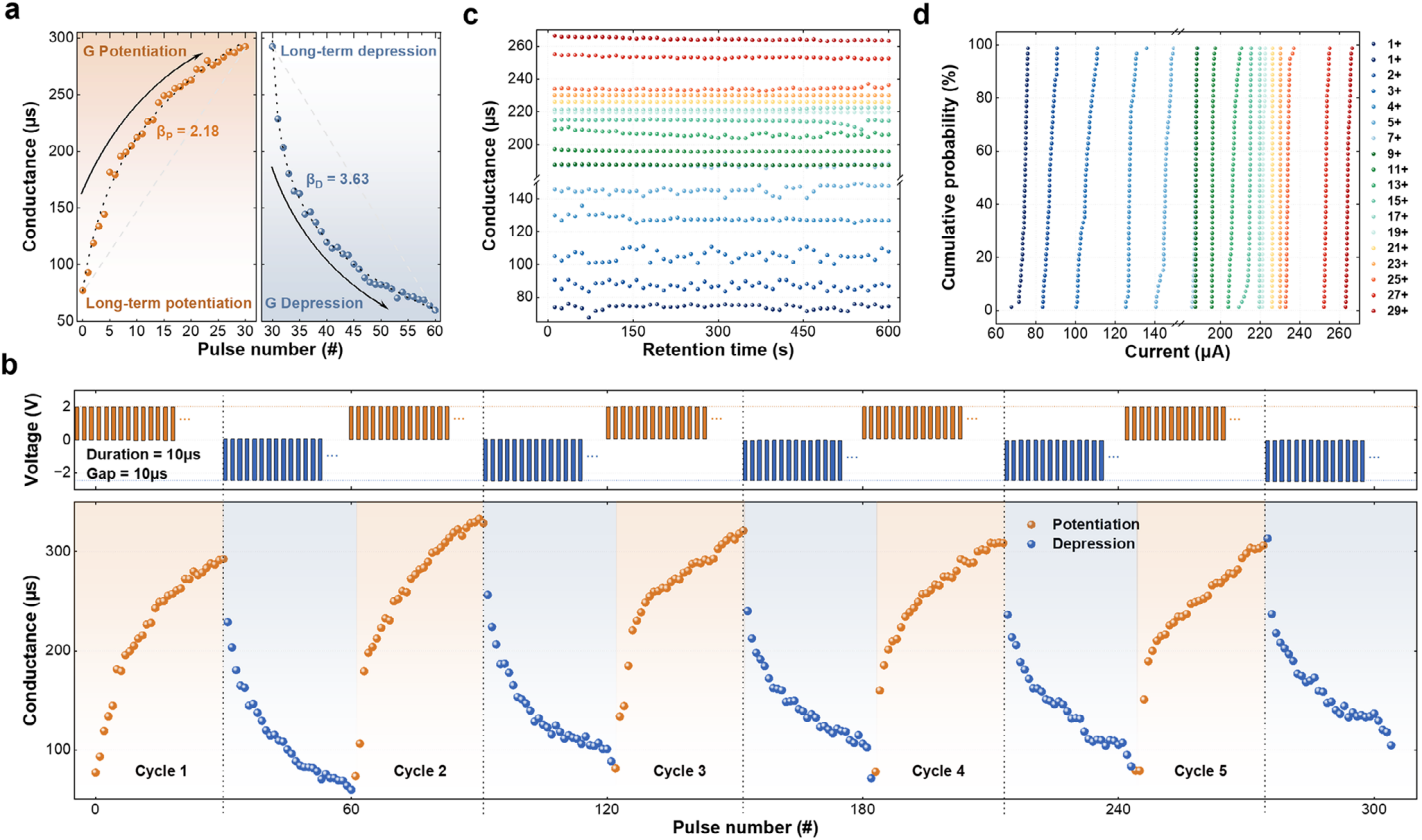
Long‐term synaptic plasticity and memory retention in the nanoporous SiO_2_ memristor. (a) Long‐term potentiation (LTP) and long‐term depression (LTD) triggered by sequential trains of positive and negative pulses, showing smooth, analog‐like conductance modulation. (b) Multiple LTP/LTD cycles demonstrating stable and reversible conductance updates. (c) Retention characteristics of programmed conductance states measured over 600 s, confirming robust memory stability. (d) Cumulative probability distributions of the retained conductance states, exhibiting tightly clustered distributions and robust state stability.

Beyond short‐term synaptic responses, the nanoporous SiO_2_ memristor can be reconfigured from short‐term to long‐term plasticity (LTP) through CC modulation, further demonstrating the device's versatility in emulating diverse neural learning processes. This tunability allows the same physical device to gradually transition from volatile, short‐lived responses to stable, nonvolatile conductance states, closely mimicking the biological progression from temporary potentiation to consolidated memory formation. When operated under a higher compliance current, the device transitioned into a nonvolatile bipolar switching regime. In this mode, 30 positive pulses (2 V, 1 µs width, 1 µs gap) were applied to induce potentiation, followed by 30 negative pulses (−2.5 V, 1 µs width, 1 µs gap) to trigger depression (Figure 6a). The conductance increased and decreased systematically with each pulse, demonstrating gradual analog LTP/LTD characteristics. The evolution of conductance during potentiation and depression follows an exponential weight‐update rule, described by [63]:

(5) $$G_{n+1}=G_{n}+\Delta G=G_{n}+\alpha_{P}e^{-\beta_{P}\frac{G_{n}-G_{min}}{G_{max}-G_{min}}}\;\left(\Delta G>0,\;G↑\right)$$

(6) $$G_{n+1}=G_{n}\;-\Delta G=G_{n}\;-\alpha_{D}e^{-\beta_{D}\frac{G_{max}-G_{n}}{G_{max}-G_{min}}}\left(\Delta G<0,\;G↓\right)$$

where *G*_*n* + 1_ and *G_n_* represent the conductance values after the (*n* + 1)^th^ and *n* ^th^ pulses, respectively, and α and β denote the magnitude and nonlinearity of conductance modulation. The extracted nonlinearity indices for LTP and LTD are 2.18 and 3.63, respectively. Based on the measured conductance range and the applied pulse condition (1 V, 10 µs), the energy consumption for each long‐term synaptic update event was estimated to be ca. 1.75 nJ per pulse, further confirming the energy‐efficient operation of the device during long‐term plasticity modulation. Repeated potentiation/depression cycling (Figure 6b) demonstrates stable analog conductance modulation during repeated pulse operation. To further quantitatively evaluate the reproducibility, dynamic range, and update precision of the analog weight modulation, statistical analysis of the LTP/LTD fitting parameters was performed over five repeated update cycles based on the exponential conductance‐update model described in Equations (5) and (6). The extracted fitting parameters are summarized in Table S2. Dynamic range (*G_max_*/*G_min_* = 4.37 ± 0.41), nonlinearity parameters (*β_P_* = 3.09 ± 0.64 and *β_D_* = 4.20 ± 0.40), and fitting coefficients (average R^2^ > 0.95) exhibited relatively small cycle‐to‐cycle variations, indicating acceptable update reproducibility and stable analog weight‐update characteristics during repeated pulse training. Measurements from three devices (Figure S14) further confirm device‐to‐device uniformity, exhibiting similar LTP/LTD characteristics and dynamic ranges. The device also maintained its programmed conductance states for over 600 s (Figure 6c), confirming robust long‐term memory retention. Figure 6d further highlights the stabilization of memory states with cumulative potentiation, with detailed statistical analysis (σ/µ) provided in Table S3. These results demonstrate that the nanoporous SiO_2_ memristor supports CC‐tunable LTP/LTD, stable multilevel memory retention, and consistent device‐to‐device analog behavior. This intrinsic adaptability establishes a solid foundation for implementing in‐hardware learning and readout layers in physical reservoir computing architectures.

### 2.3 Reservoir Computing With Dual‐Mode Memristors

**FIGURE 7:**
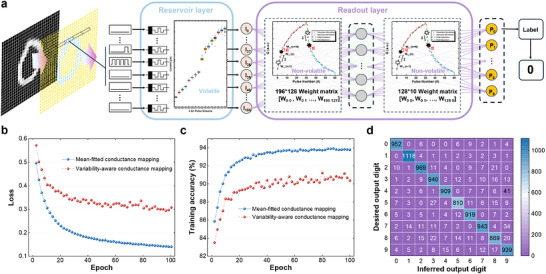
Benchmark demonstration of MNIST handwritten‐digit classification using a memristor‐based reservoir computing (RC) system. (a) Schematic of the hardware RC architecture, where short‐term plasticity‐driven reservoir dynamics are coupled with a long‐term plasticity‐trained readout layer, both realized using cross‐point‐structured nanoporous SiO_2_ memristors. (b) Evolution of quantized‐state loss during network training under mean‐fitting (blue) and variability‐aware (red) conditions. (c) Training accuracy of the memristive reservoir‐computing system as a function of training epoch under mean‐fitting (blue) and variability‐aware (red) conditions. (d) Confusion matrix obtained under the mean‐fitting condition for 10 000 MNIST test images, confirming accurate recognition across all digit categories.

The unique capability of the nanoporous SiO_2_ memristor to switch between short‐term and long‐term plasticity modes enables the realization of a hardware‐compatible RC system within a single material platform. This dual‐mode operation eliminates the need for heterogeneous devices, allowing the same memristor crossbar to serve as both the dynamic reservoir and the adaptive readout layer. To benchmark such dual‐mode computing capability, we first implemented a hardware RC system for MNIST handwritten‐digit classification, a standard test used to benchmark the performance of different neuromorphic computing platforms. This demonstration validates the network's functionality before extending it to real‐world temporal data such as biosignals. As shown in Figure 7a, the RC system is constructed on cross‐point devices of Ag/nSiO_2_/TiN memristors, in which both the reservoir and readout layers are realized using the same memristor platform. Each 28 × 28‐pixel MNIST image is binarized and segmented into 196 four‐bit groups, which are directly encoded into voltage‐pulse sequences (1 V, 10 ms width, 0.5 ms gap) and applied to the nanoporous SiO_2_ memristor. Owing to its intrinsic short‐term plasticity and nonlinear transient dynamics, the device maps these digital inputs into a high‐dimensional analog feature space, enabling the extraction of spatiotemporal correlations between neighboring pixel patterns [9]. As shown in Figure S15a, all sixteen possible four‐bit input states are distinctly separated, confirming the richness of the reservoir state space. The corresponding experimental transient responses (Figure S15b) further demonstrate that each 4‐bit sequence (0000–1111) produces a distinct and reproducible current trajectory, with excellent endurance over 100 repeated cycles.

The readout layer was implemented as a three‐layer neural network comprising 196 input nodes, 128 hidden neurons, and 10 output neurons (corresponding to digits 0–9). In this stage, the same nanoporous SiO_2_ memristors were operated in their long‐term plasticity mode to store and update synaptic weights through LTP and LTD mechanisms. Weight matrices of size 196 × 128 and 128×10 were trained via backpropagation to minimize cross‐entropy loss during supervised learning. The conductance updates followed the experimentally measured exponential rule (Equations (5) and (6)), while the weights were further constrained to discrete conductance states through a periodic quantization scheme, as detailed in the *Experimental Section*. The network was trained and tested using the standard MNIST dataset, comprising 60 000 training and 10 000 test images. Full experimental procedures are provided in the *Experimental Section*, and the evolution of hidden‐layer weight updates is shown in Figure S16. The memristive reservoir‐computing system was first evaluated under the mean‐fitting condition, where conductance updates were described using the experimentally fitted average LTP/LTD characteristics. Under this condition, the network exhibited stable convergence, with the quantized‐state loss steadily decreasing (Figure 7b, blue) and the training accuracy increasing (Figure 7c, blue) during training. A corresponding test accuracy of 93.7% was achieved, as shown in the confusion matrix in Figure 7d, confirming that the experimentally derived conductance‐update model can support reliable MNIST classification. To further assess the influence of realistic device variation, stochastic conductance‐update fluctuations were then incorporated into the training process based on the experimentally extracted standard deviations (SDs) of the fitting parameters obtained from repeated LTP/LTD cycles (Table S2). During each training epoch, a new set of fitting parameters was randomly sampled within the measured variation range to emulate cycle‐to‐cycle conductance fluctuations during conductance‐state projection (“snapping”). As shown in Figure 7b,c (red), the inclusion of stochastic variation introduces increased loss fluctuations during training and leads to a moderate reduction in classification accuracy from 93.7% to approximately 90.4% (the corresponding confusion matrices are shown in Figure 7d and Figure S17). Nevertheless, the network still maintains stable convergence under the variability‐aware condition, indicating that the reservoir‐computing framework remains tolerant to realistic conductance nonlinearity, update variation, and discretization effects.

Table 1 summarizes representative memristor‐based neuromorphic systems for MNIST classification. Most reported systems exploit only a single memristive functionality, relying exclusively on either STP for dynamic processing or LTP for weight storage. Only a few studies integrate both functions, typically requiring heterogeneous materials or distinct device types. In contrast, the system presented here integrates both reservoir dynamics and readout functionality within a single nanoporous SiO_2_ material platform, leveraging STP for temporal feature encoding and LTP for stable synaptic weight storage in the same device architecture. Despite this simplified, single‐material approach, the resulting hardware reservoir computing system achieves competitive classification performance. This intrinsic dual‐mode operation enables compact, energy‐efficient, and CMOS‐compatible neuromorphic architectures, providing a promising pathway toward scalable intelligent edge computing systems.

**TABLE 1:** Comparison of memristor‐based neuromorphic systems for MNIST classification. Summary of reservoir and readout configurations, reservoir implementation, and reported accuracies from different works.

Reservoir device	Readout device	Reservoir implementation	Readout training	Accuracy	Ref
Pd/Au/WO_x_/W/SiO_2_/Si	N/A	Physical	Offline (software)	91.1%	[38]
TiN/HAO/ZrO_2_/n^+^‐Si	N/A	Physical	Offline (software)	93.70%	[64]
Pt/GDC/CeO_2_/Pt	N/A	Physical	Offline (software)	90.49%	[65]
Ag/Bi_2_SeO_5_/Au	N/A	Physical	Offline (software)	97.03%	[66]
ITO/p‐NiO/n‐IGZO/Ti/Pt	N/A	Physical	Offline (software)	95.20%	[67]
ITO/HfAlO_x_/n^+^‐Si	N/A	Physical	Offline (software)	96.37%	[68]
N/A	Pt/Ga_2_O_3_/Pt	N/A	Offline (software)	85.30%	[69]
N/A	Ag/WTe_2_/Pt	N/A	Offline (software)	92%	[70]
N/A	Ag/CdS/CZTSSe/Mo	N/A	Offline (software)	94.10%	[71]
N/A	TiN/TiO_2_/BaTiO_3_/Pt	N/A	Offline (software)	94.70%	[72]
N/A	Au/MoS_2_/Au	N/A	Offline (software)	86.73%	[73]
Cu/h‐BN/Au	Cu/a‐BN/Au	Physical	Offline (software)	92.70%	[74]
Au/SiO_2_/CoO_x_/ITO	Au/SiO_2_/CoO_x_/ITO	Physical	Offline (software)	93.40%	[75]
Pb/SiO_x_:Ag/Pt/Ti/Si	Pd/Ta_2_O_5_/Ta and 1T1R	Circuit‐level	On‐chip / in‐situ	83%	[13]
Ag/nanoporous SiO_2_/TiN	Ag/nanoporous SiO_2_/TiN	Physical	Offline (software)	**93.7%**	**This work**

### 2.4 Temporal Biosignal Processing

Following the benchmark demonstration with the MNIST dataset, we further evaluated the practical applicability of the dual‐mode nanoporous SiO_2_ memristor by performing electrocardiogram (ECG) signal classification that requires temporal sensitivity and dynamic information encoding. ECG signals are inherently time‐dependent, recorded as voltage waveforms that reflect the rhythmic electrical activity of the heart's conduction system [76]. Each cardiac cycle comprises distinct components: the P wave, representing atrial depolarization; the QRS complex, corresponding to ventricular depolarization and contraction; and the T wave, denoting ventricular repolarization and recovery [77]. Accurate identification of variations in these features enables the detection of cardiac abnormalities such as arrhythmias, ischemia, or conduction disorders.

**FIGURE 8:**
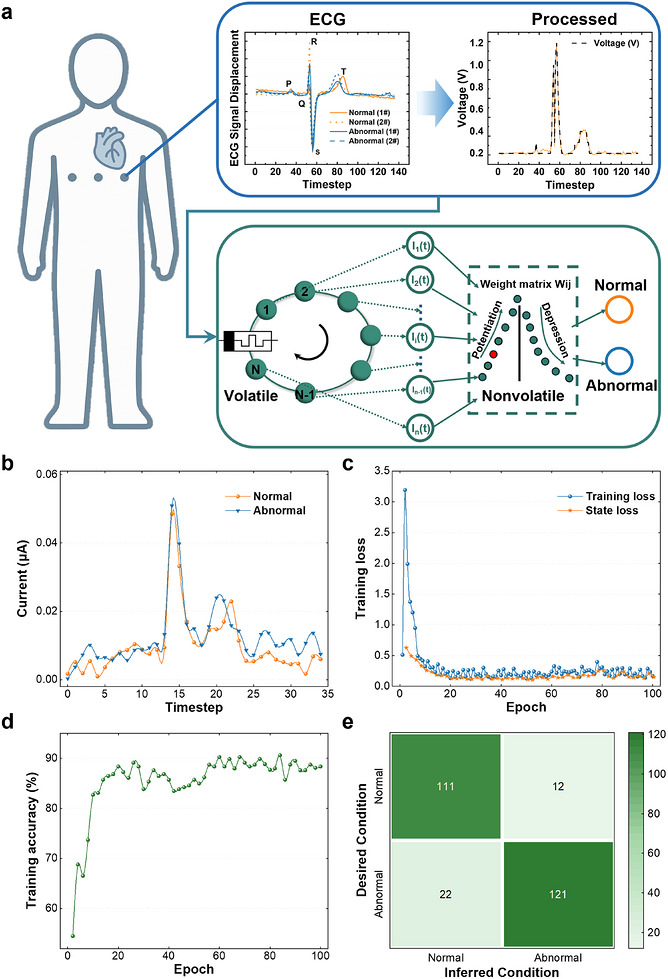
ECG signal classification using the nanoporous SiO_2_ memristor‐based RC system. (a) Schematic illustration of the RC framework, where STP‐based virtual nodes form the reservoir and a LTP‐trained readout layer performs classification. (b) Current response profiles of 34 virtual nodes for two heart conditions: normal (red solid lines) and abnormal (blue dashed lines). (c) Evolution of training loss and state loss (evaluated every 2 epochs) as a function of training epochs. (d) Training accuracy of the RC system during ECG classification as a function of training epochs. (e) Confusion matrix of the classification results for 266 ECG signals.

To capture these temporal dynamics, a memristor‐based neuromorphic architecture was developed (Figure 8a). The ECGFiveDays dataset from the UCR Time Series Classification Archive was used, consisting of 618 training and 266 testing samples collected over five days from a single subject [78]. Each sample contains 136 time steps, and the classification task distinguishes between two heartbeat morphologies corresponding to normal and abnormal cardiac patterns. In this implementation, the nanoporous SiO_2_ memristor was operated in its volatile (STP) mode. Raw ECG signals were first converted to absolute values and normalized within the range [0.2, 1.2] V, then used to generate programming pulse sequences for device input encoding. Leveraging the device's nonlinear transient dynamics and short‐term memory, internal conductance states were sampled at fixed intervals during each input sequence to create virtual nodes—each representing a distinct temporal snapshot of the reservoir's response [9, 78].

The collection of these virtual node currents constitutes a high‐dimensional feature space capable of mapping subtle temporal variations in the ECG waveforms. For this study, the input sequence was time‐multiplexed by sampling every four‐time steps, yielding 34 virtual nodes. Figure 8b shows representative current responses for normal and abnormal heartbeats, demonstrating clear distinctions in the reservoir dynamics. These node outputs were subsequently processed by a software‐implemented readout network based on the experimentally measured conductance‐update characteristics of the same nanoporous SiO_2_ memristor operated in the nonvolatile (LTP) mode, following the workflow detailed in the *Experimental Section*. The hidden‐layer weight‐update process is shown in Figure S18. The training and quantized state losses (Figure 8c) decreased steadily across epochs, confirming stable learning behavior.

The system's classification performance is summarized in Figure 8d,e. The trained RC network achieved test accuracy exceeding 88%, successfully identifying 111 normal and 121 abnormal cases. The confusion matrix confirms reliable separation between the two classes, demonstrating that the memristor‐based reservoir efficiently encodes and distinguishes complex temporal features in biosignals. Consistent with the MNIST results, the system remains robust to conductance quantization across different tasks. These results highlight the potential of the nanoporous SiO_2_ memristor as a compact, energy‐efficient platform for neuromorphic signal processing and hardware‐assisted biosignal analysis applications.

Beyond the demonstrated device‐level performance, it is important to consider the scalability of the proposed architecture. Although the present demonstrations were performed using cross‐point structured memristors, the proposed architecture is inherently compatible with large‐scale crossbar‐array implementation for scalable neuromorphic integration. In such systems, non‐ideal effects, including sneak‐path currents, line resistance, and interconnect parasitics, may affect switching uniformity and readout accuracy [79, 80, 81]. These challenges can be mitigated through established strategies such as selector integration and optimized biasing schemes. Leveraging its forming‐free operation, tunable dual‐mode switching characteristics, and CMOS compatibility, the nanoporous SiO_2_ memristor platform offers a promising pathway toward scalable neuromorphic hardware.

## 3 Conclusions

In summary, we have developed a cross‐point nanoporous SiO_2_ memristor that unifies volatile and nonvolatile switching behaviors within a single material system, enabling tunable short‐term and long‐term plasticity in the same device architecture. This dual‐mode functionality, controlled simply through compliance‐current modulation, allowed both dynamic reservoir encoding and adaptive weight storage to be realized on one hardware platform, reducing the reliance on multi‐material integration. Leveraging this capability, we implemented a hybrid memristor‐based reservoir computing system framework using experimentally measured nanoporous SiO_2_ device characteristics. The system achieved approximately 93.7% accuracy in MNIST handwritten‐digit recognition, while maintaining competitive performance compared with previously reported memristor‐based neuromorphic systems and further demonstrated robust ECG temporal biosignal classification with an accuracy above 88%. These results establish the nSiO_2_ cross‐point as a scalable, CMOS‐compatible, and energy‐efficient neuromorphic platform capable of both transient and persistent information processing, providing a promising pathway toward integrated and adaptive edge neuromorphic hardware. Future efforts will focus on scalable array integration and fully hardware‐compatible training schemes, paving the way toward practical memristive systems for temporal information‐processing applications.

## 4 Experimental Section

### 4.1 Synthesis of Nanoporous Silica Films

Triblock copolymer Pluronic F‐127 (Mw = 12600; PEO_106_PPO_70–_PEO_106_), tetraethyl orthosilicate (TEOS), and 37% hydrochloric acid (HCl) were purchased from Sigma–Aldrich Company Ltd, and no further purification was required when using them. A 1 M HCl solution was prepared by diluting the concentrated HCl with deionized water. Ethanol (≥ 99.8%) and dichloromethane (DCM) were obtained from Fisher Scientific. The precursor solution was prepared via the evaporation‐induced self‐assembly (EISA) method. Specifically, 1 g of TEOS, 5.64 g of ethanol, 0.8 g of deionized water, and 0.1 g of 1 M hydrochloric acid were mixed and stirred at 338 K for 45 min. Separately, 0.3024 g of Pluronic F‐127 was dissolved into 5.64 g of ethanol under ambient conditions. Subsequently, the two solutions were combined and stirred thoroughly at room temperature for 60 min. The resulting precursor solution had molar ratios of TEOS: F‐127: HCl: H_2_O: EtOH of 1: 0.005: 0.021: 9.2: 51.

The nanoporous silica films were deposited onto patterned TiN/SiO_2_/Si substrates by spin coating at 3000 rpm for 60 s inside a humidity‐controlled chamber (75% RH, 298 K) using an Ossila vacuum‐free coater. The coated films were aged in the same chamber for 72 h (75% RH), followed by thermal treatment at 120°C for 10 h. Surfactant removal was achieved by immersion in DCM for 4 h, followed by calcination at 350°C for 5 h. The resulting films are referred to as nanoporous silica films.

### 4.2 Memristor Device Fabrication and Characterization

6‐inch SiO_2_/Si wafers were spin‐coated with photoresist AZ2070 and patterned using an EVG620TB Mask Aligner, followed by development in AZ726 MIF. A 40 nm TiN bottom electrode was deposited via reactive sputtering (Leybold Helios Pro XL system, 3 kW RF power, Ti target, N_2_ atmosphere). The first lift‐off was performed using an SSE Mask Cleaner and Lift‐Off Tool with N‐methyl‐2‐pyrrolidone (NMP) heated to 60°C at 1.5 bar for 30 min to define bottom electrodes. The wafers were diced into 20 mm × 20 mm chips prior to film deposition. After the nSiO_2_ coating, photoresist S1813 was applied for the second lithography and developed in MF319. The exposed nSiO_2_ layer on electrode pads was removed by reactive ion etching (RIE) using Ar/CHF_3_ gases (505 V DC bias, 203 W forward power, 4 W reflected). Following photoresist removal, AZ2070 was used again for top‐electrode patterning. After development, 200 nm Ag was deposited via e‐beam evaporation (Leybold Lab 700 system), followed by a final lift‐off to form the Ag/SiO_2_/TiN cross‐point memristors.

Film thickness was measured using a Woollam M‐2000 XI spectroscopic ellipsometer. High‐resolution SEM (Zeiss Sigma 500 FESEM) was conducted at 1.75 kV accelerating voltage, 2 mm working distance, and 200 000× magnification using an InLens Duo detector. GISAXS patterns were collected on a Rigaku SmartLab equipped with a HyPix‐3000 detector (λ = 1.54 Å, incident angle = 0.3°, sample–detector distance ≈ 300 mm). Data were analyzed using the GIXSGUI MATLAB toolbox. Electrical characterization was performed at room temperature using a Keysight B1500A semiconductor parameter analyzer connected to a probe station. All measurements were conducted in ambient air unless stated otherwise.

### 4.3 Reservoir Computing Training

The system is implemented in a hybrid hardware–software framework, in which the reservoir layer is physically realized using the intrinsic device dynamics, while the readout layer is trained offline and mapped onto experimentally accessible conductance states.

For MNIST handwritten‐digit recognition, the readout network comprised three sequential layers: an input layer with a shape of 196, a hidden dense layer with 128 neurons activated by ReLU, and an output dense layer with 10 neurons employing softmax activation to produce class probabilities. The two weight matrices of sizes—196 × 128 and 128 × 10—were initialized using He uniform distribution. The model was then compiled with the Adam optimizer (learning rate = 0.001), using sparse categorical cross‐entropy as the loss function, and accuracy was monitored as an additional metric during training.

For the ECG recognition, the first layer was a 1D convolutional layer with 64 filters, a kernel size of 3, and ReLU activation, designed to extract local temporal features. This was followed by a max pooling layer with a pool size of 2 to reduce the feature map dimensions. The pooled features were then flattened into a vector and passed through a dense fully connected layer with 128 neurons and ReLU activation, serving as the hidden layer for complex feature learning. The output layer utilized softmax activation with two output neurons corresponding to normal and abnormal classes. The model was also trained using the Adam optimizer with a categorical cross‐entropy loss function.

To emulate memristor behavior, the synaptic weights were constrained to empirically measured conductance states normalized to the range [‐0.5, 0.5]. Weight evolution followed the experimentally fitted potentiation and depression characteristics described by Equations (5) and (6), thereby incorporating conductance nonlinearity, update asymmetry, and discretization effects into the learning process. During each training epoch, the gradient of the loss function with respect to each trainable parameter was calculated as:

(7) $$Gradient=\frac{\partial Loss}{\partial Weight}\;$$

Depending on the sign of the gradient, the weights were updated to model potentiation or depression: if the gradient was negative, the conductance (weight) was increased (Δ*G* > 0); if positive, it was decreased (Δ*G* < 0). To maintain physical stability and prevent drift, a state “snapping” quantization scheme was adopted, whereby the weights were periodically projected to the nearest experimentally accessible conductance state every N training epochs (every 2 epochs in this work).

## Conflicts of Interest

The authors declare no conflicts of interest.

## Supporting information




**Supporting File**: advs76163‐sup‐0001‐SuppMat.pdf.

## Data Availability

The data that support the findings of this study are openly available in University of Southampton repository at https://eprints.soton.ac.uk/, reference number D3940.
