# Supplementary material for: Dual‐Mode Nanoporous SiO2 Memristors with Coexisting Volatile and Nonvolatile Dynamics for Reservoir Computing
Source: Adv Sci (Weinh). 2026 Jun 18;13(51):e76163. doi: 10.1002/advs.76163 (PMC13336861; doi:10.1002/advs.76163)
Supplement: Supplementary file 1 [file ADVS-13-e76163-s001.pdf]

Supporting Information

Dual-Mode Nanoporous SiO_2 Memristors with Coexisting Volatile and Nonvolatile Dynamics for Reservoir Computing

*Bohao Ding, Tongjun Zhang, Li Shao, Nikolay Zhelev, Andrew L. Hector and Ruomeng Huang**

B. Ding, T. Zhang, R. Huang

School of Electronics and Computer Science, University of Southampton, Southampton, SO17 1BJ, UK

E-mail: **R.Huang@soton.ac.uk**

L. Shao, N. Zhelev, A. L. Hector

School of Chemistry and Chemical Engineering, University of Southampton, Southampton, SO17 1BJ, UK

Table S1. Comparison of representative dual-mode or tunable memristor platforms for neuromorphic and reservoir computing applications. V/NV denotes volatile/nonvolatile switching. STP/LTP denotes short-term plasticity/long-term plasticity

Material system	Switching strategy	DC-domain V/NV	Pulse-domain STP/LTP	Reservoir/readout implementation	Task	Ref.
**$CoFe_2O_4$**	CC-controlled	Yes	–	Device-level	–	[1]
**Sn-alloyed**	CC-controlled	Yes	–	–	–	[2]
**Hybrid oxide**	Hybrid-state-controlled	Yes	STP only	RC	MNIST	[3]
**ZnO**	CC-controlled	Yes	Yes	RC	MNIST	[4]
**SiO_2/CoO_x**	CC-controlled	Yes	Yes	Hybrid RC framework	MNIST	[5]
**Lithium-imbued TiO_x**	Ion-modulated dynamics	Yes	STP only	Hybrid RC framework	Voice recognition	[6]
**Nanoporous SiO_2**	CC-controlled	Yes	Yes	Hybrid RC framework	MNIST + ECG	This work

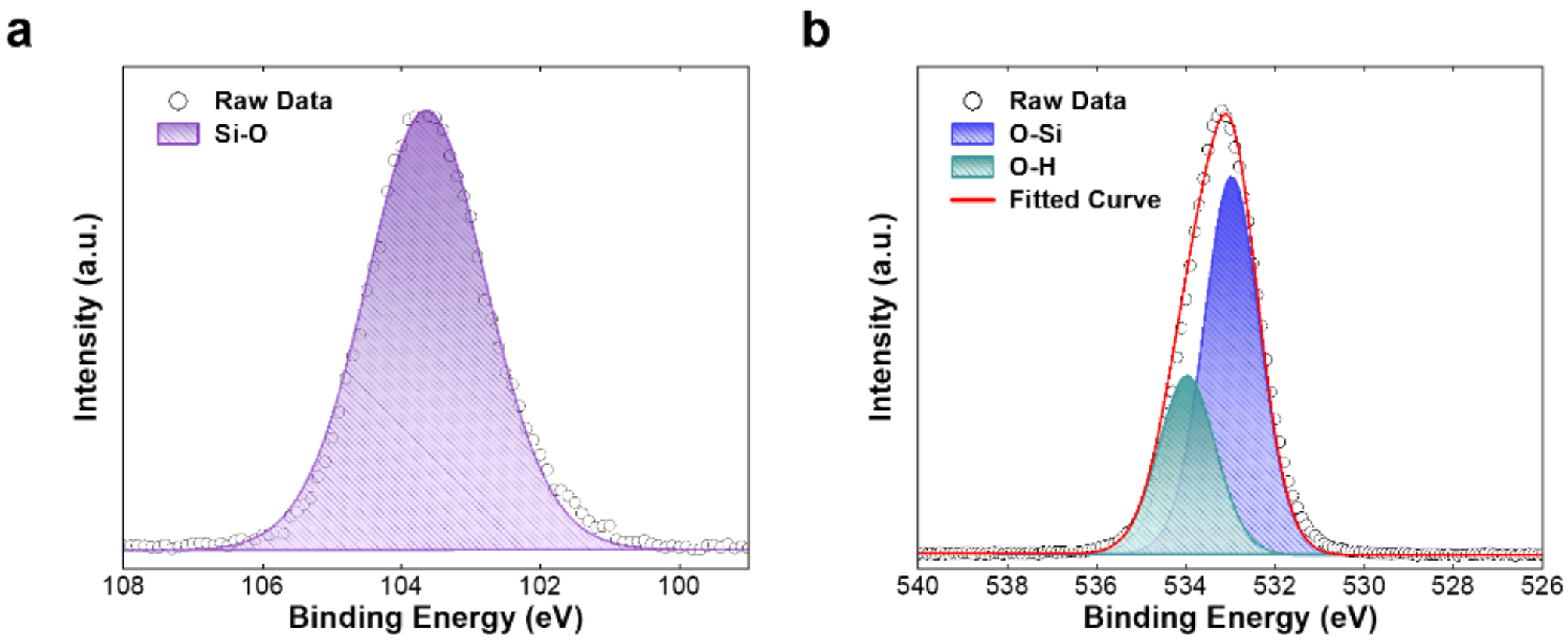

Figure S1. XPS analysis of the nanoporous SiO_2 film, showing a single peak at ~103.5 eV, characteristic of fully oxidized Si–O bonds. (a) The XPS profiles of the Si 2p core level. (b) The XPS profiles of the O 1s core level, revealing O–Si (532.9 eV) and O–H (534.0 eV) components, indicating framework oxygen and hydroxyl surface species associated with the porous silica structure.

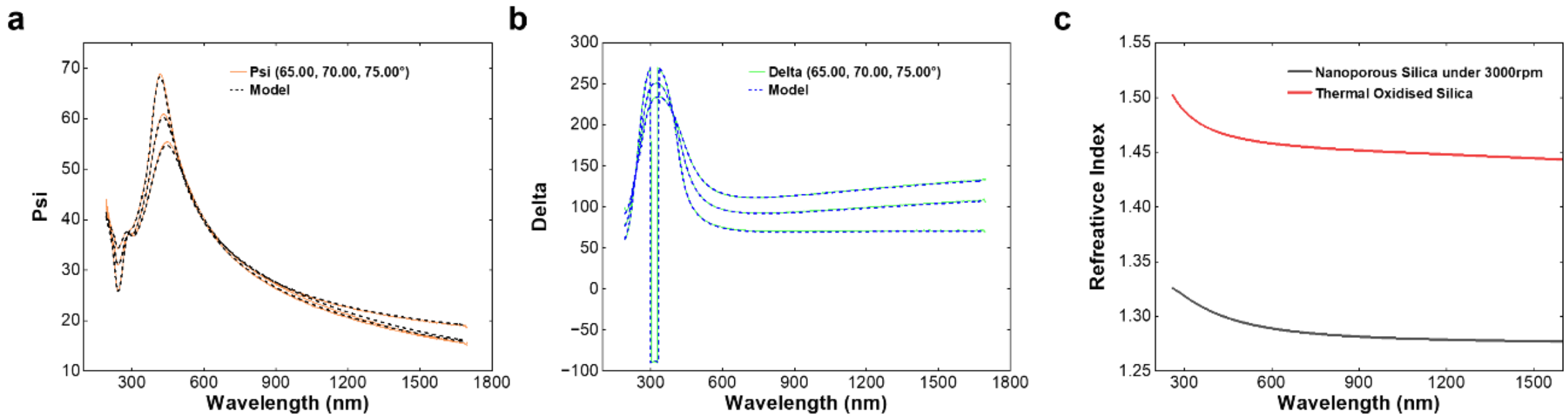

Figure S2. Ellipsometry analysis of the nanoporous SiO_2 film. (a,b) Experimental Ψ and Δ spectra fitted using a Cauchy dispersion model, yielding a film thickness of *ca.* 120 nm. (c) Refractive index dispersion of the nanoporous film compared with dense thermal SiO_2.

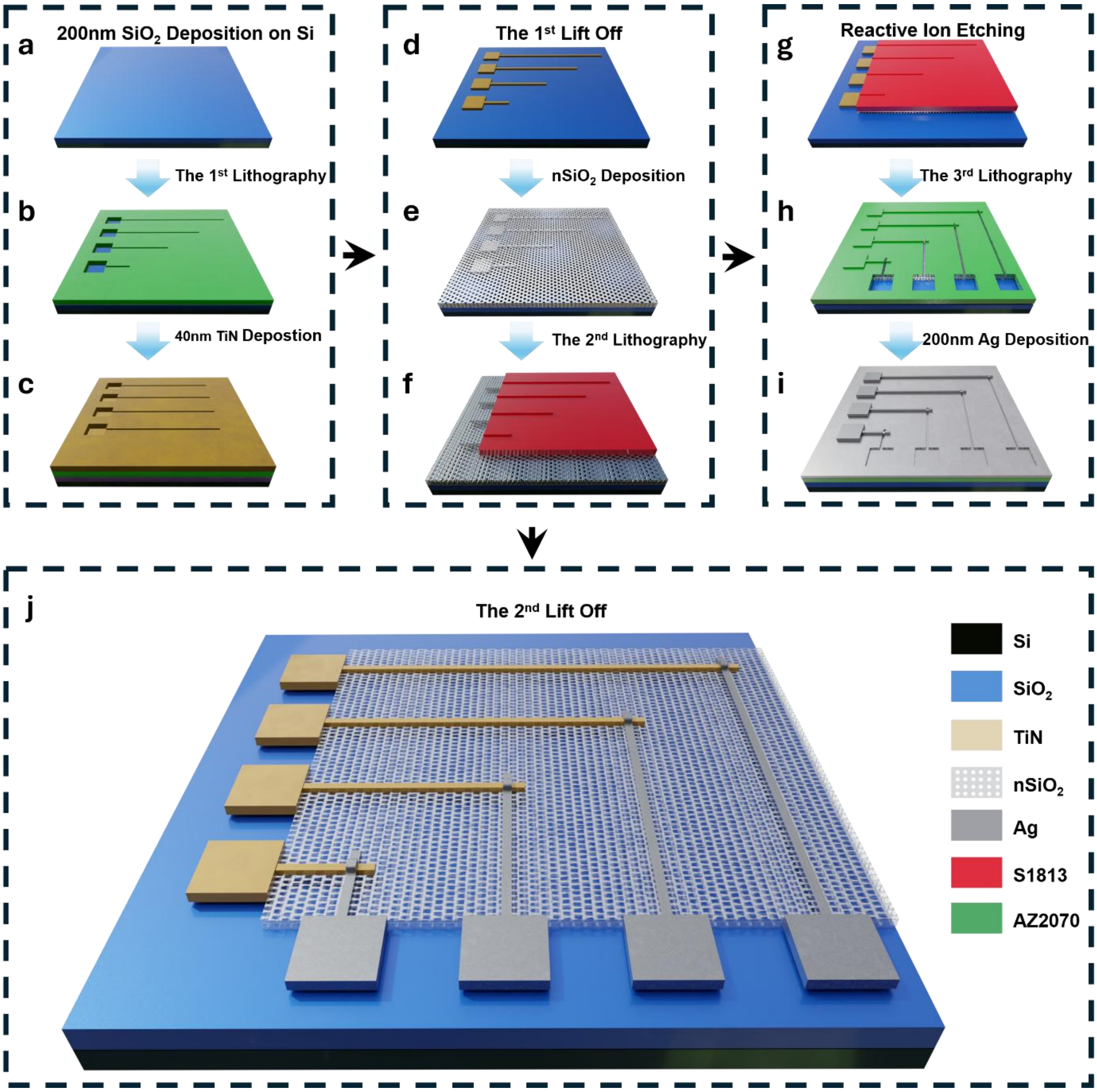

Figure S3. Schematic illustration of the fabrication process of cross-point structured nanoporous SiO_2 memristors: (a) Deposition of 200 nm SiO_2 on a Si wafer. (b) First photolithography using AZ 2070 to define the bottom-electrode pattern. (c) Deposition of 40 nm TiN. (d) First lift-off to form TiN bottom electrodes. (e) Deposition of the 120 nm $nSiO_2$ switching layer. (f) Second photolithography using S1813 to open contact windows. (g) Reactive ion etching to expose the TiN bottom electrodes. (h) Third photolithography using AZ 2070 to define the top-electrode pattern. (i) Deposition of 200 nm Ag. (j) Second lift-off to form Ag top electrodes, completing the cross-point memristors.

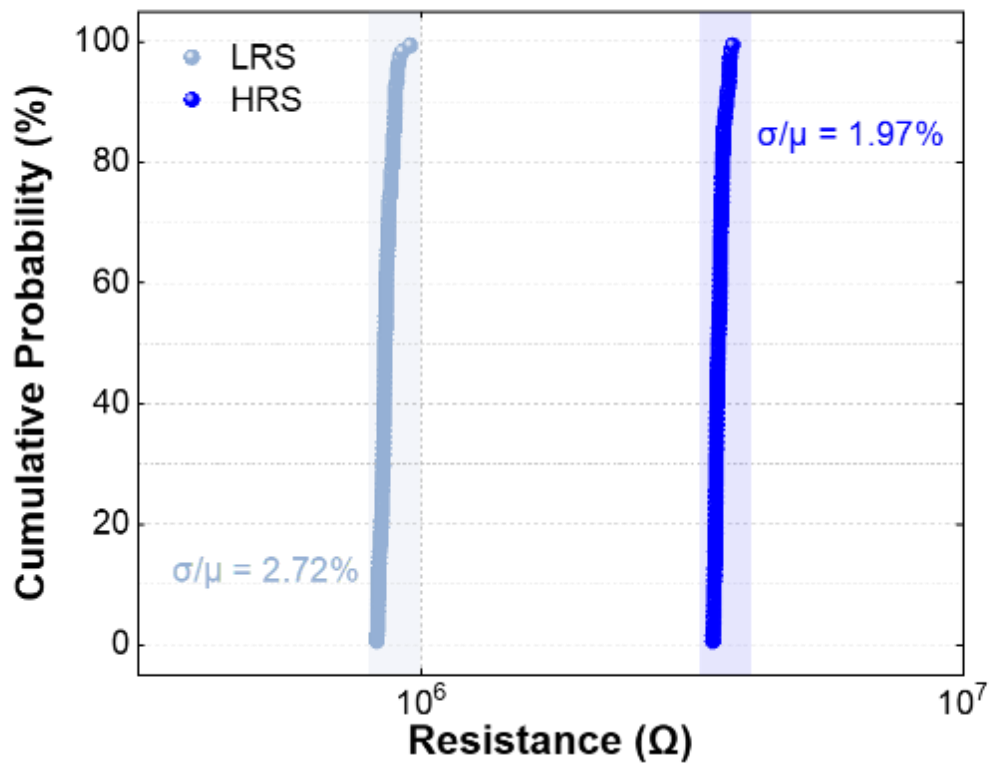

Figure S4. Cumulative probability distributions of the high-resistance state (HRS) and low-resistance state (LRS) for the nanoporous SiO_2 memristor operating in the volatile mode, demonstrating stable volatile switching behavior with limited statistical variation.

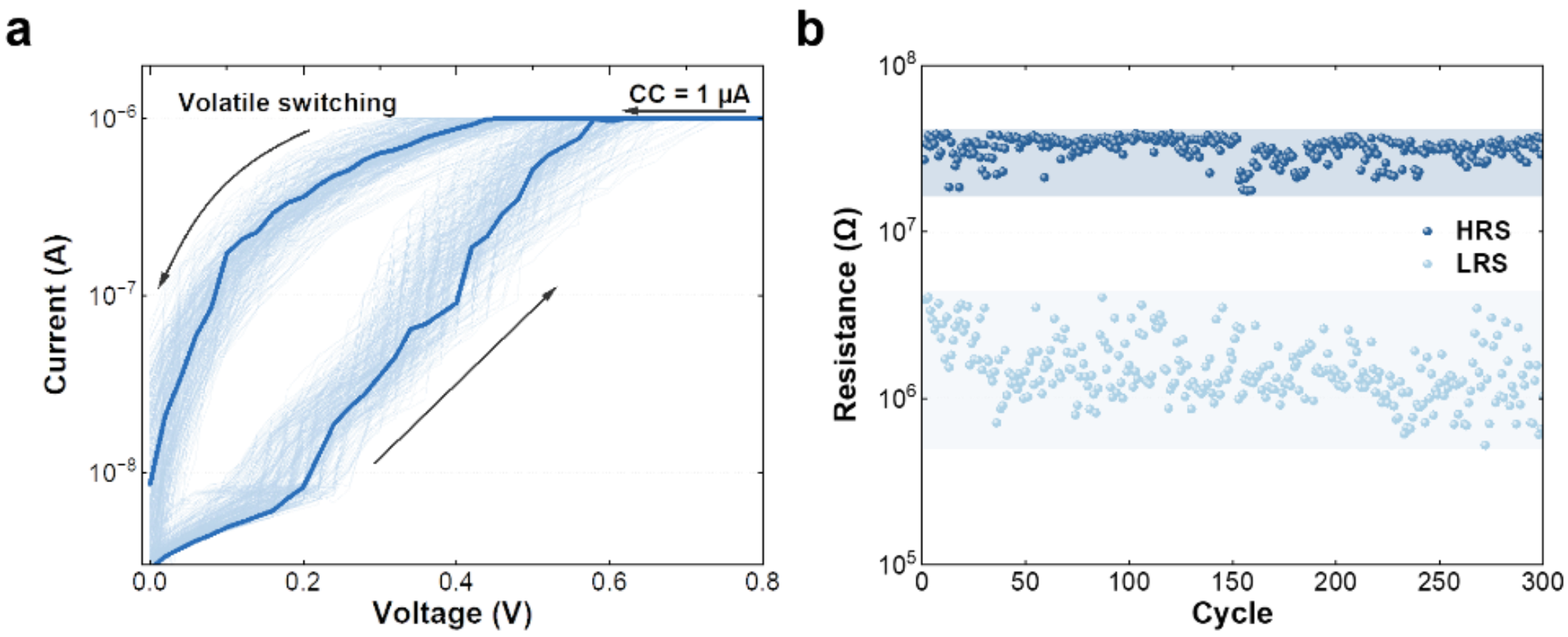

Figure S5. (a) Volatile I–V characteristics measured over 300 consecutive sweeps under a compliance current of 1 μA. (b) Corresponding endurance characteristics of the volatile mode at a 0.1 V read voltage.

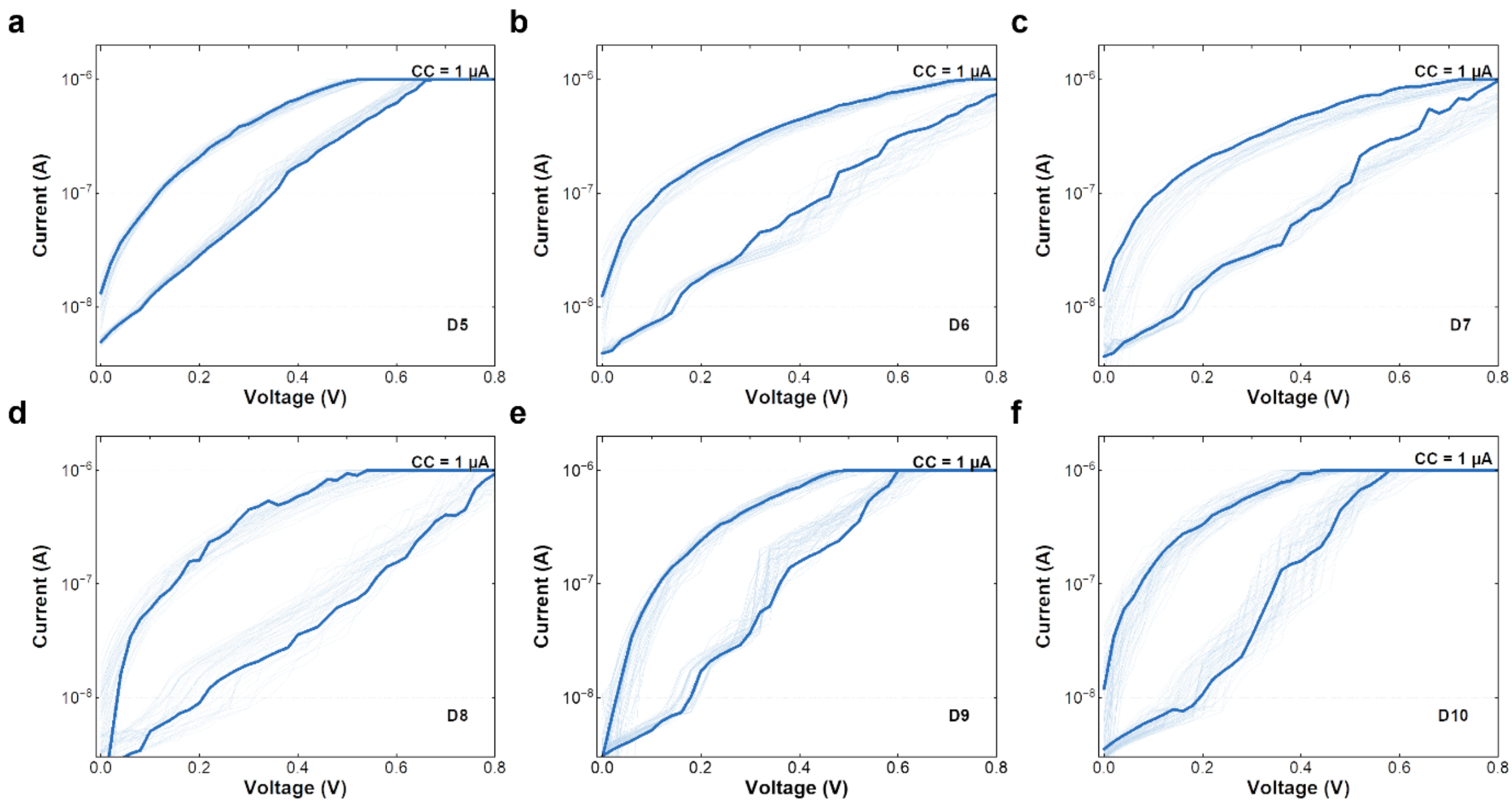

Figure S6. Statistical analysis of the volatile switching characteristics of nanoporous SiO_2 memristors. (a–f) Representative I–V curves measured from multiple devices (D5–D10), each tested over 50 consecutive switching cycles, showing consistent switching behavior across devices.

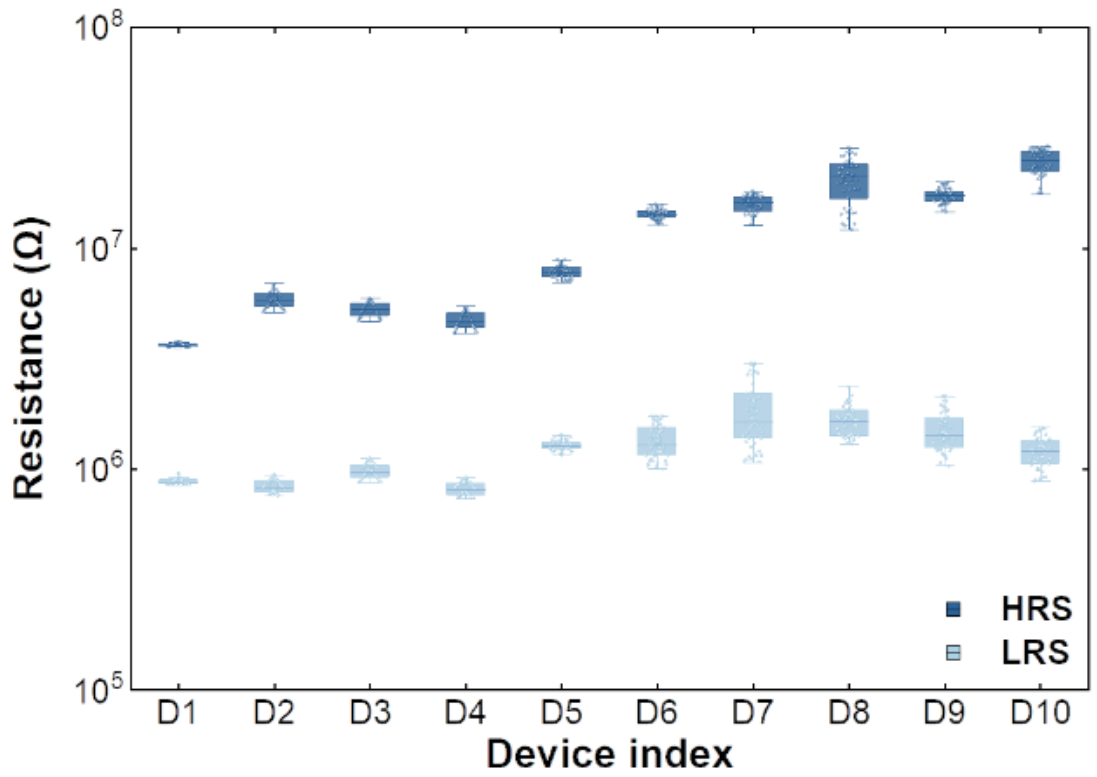

Figure S7. HRS/LRS resistance distributions measured from multiple devices (D1–D10) under the volatile switching mode.

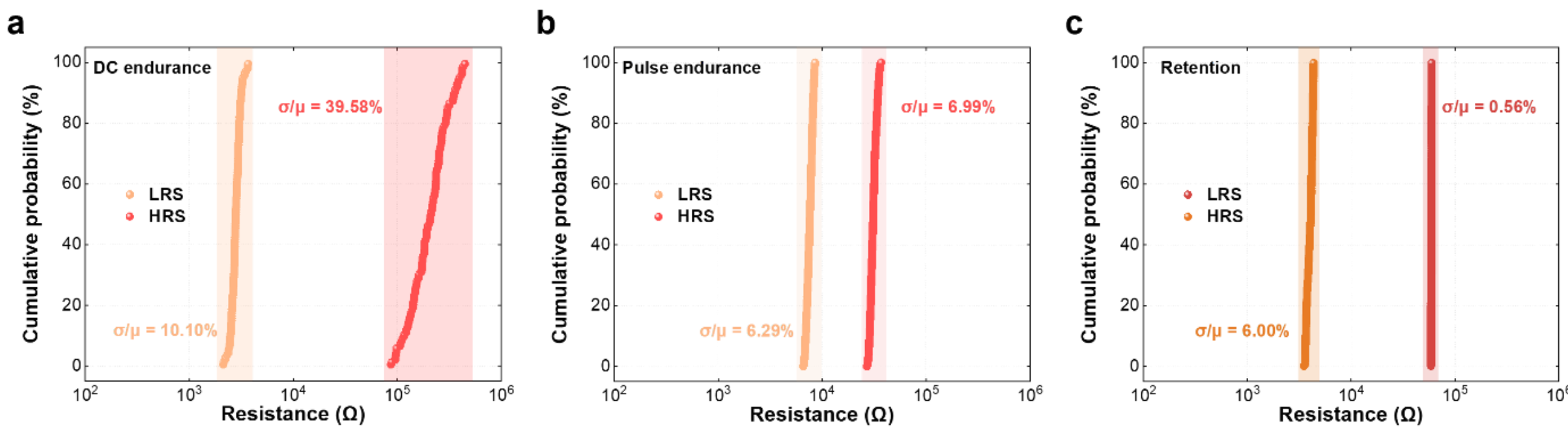

Figure S8. Distributions of the high-resistance state (HRS) and low-resistance state (LRS) measured from the nonvolatile switching mode of the nanoporous SiO_2 memristor for (a) DC sweep endurance, (b) pulse endurance measurements, and (c) retention. The corresponding coefficients of variation (σ/μ) are labeled for each resistance state.

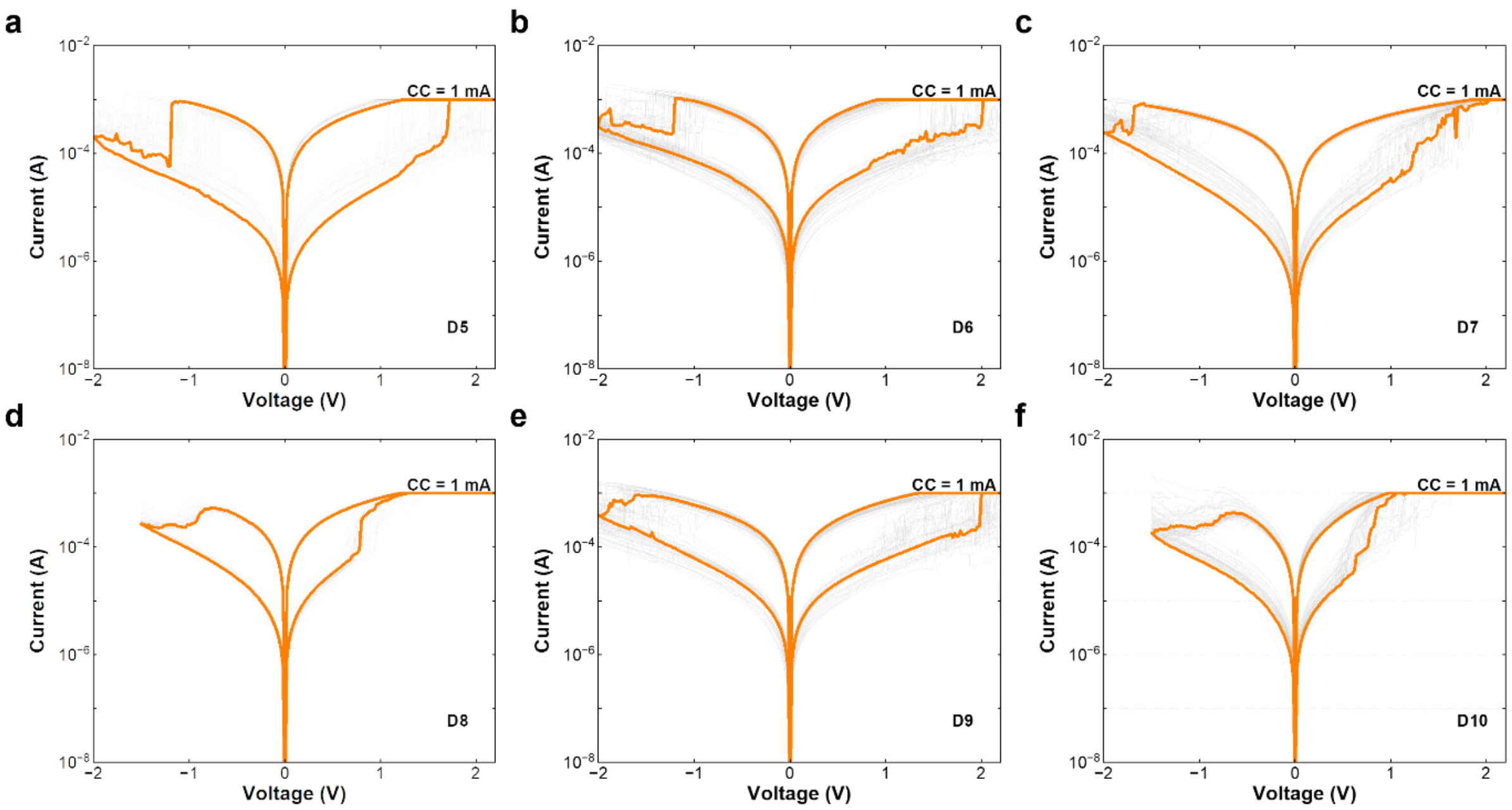

Figure S9. Statistical analysis of the nonvolatile switching characteristics of nanoporous SiO_2 memristors. (a–f) Representative I–V curves measured from multiple devices (D5–D10), each tested over 50 consecutive switching cycles, showing consistent switching behavior across devices.

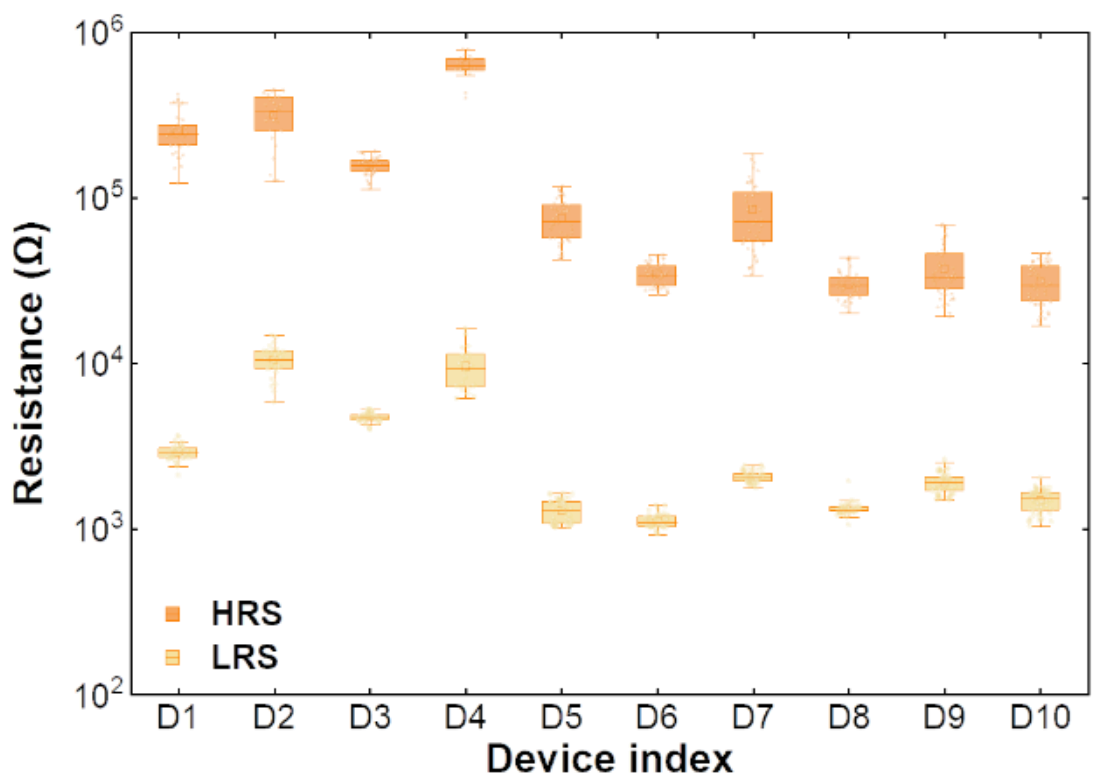

Figure S10. HRS/LRS resistance distributions measured from multiple devices (D1–D10) under the nonvolatile switching mode.

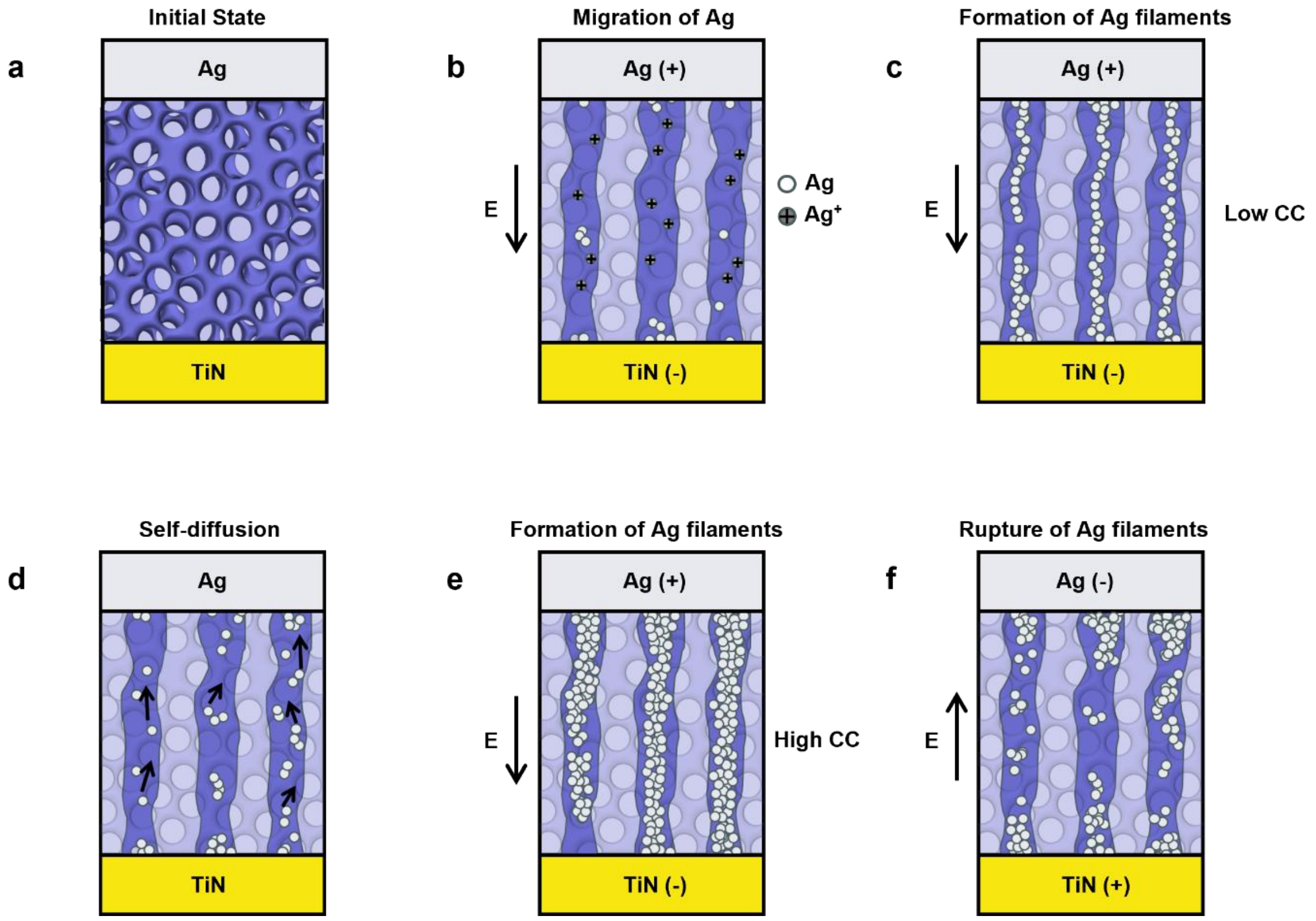

Figure S11. Proposed switching mechanism of the cross-point structured nanoporous SiO_2-based memristor. (a) Initial state of a nanopore inside the device. (b) Migration of Ag^+ ions driven by the electric field under a low compliance current (CC). (c) Formation of Ag filaments under a lower CC during the SET process. (d) Spontaneous diffusion of filaments once the external stimulus is removed, accounting for volatile switching. (e) Formation of more robust filaments under a higher CC during the SET process. (f) Rupture of Ag filaments during the RESET process induced by applying a negative bias.

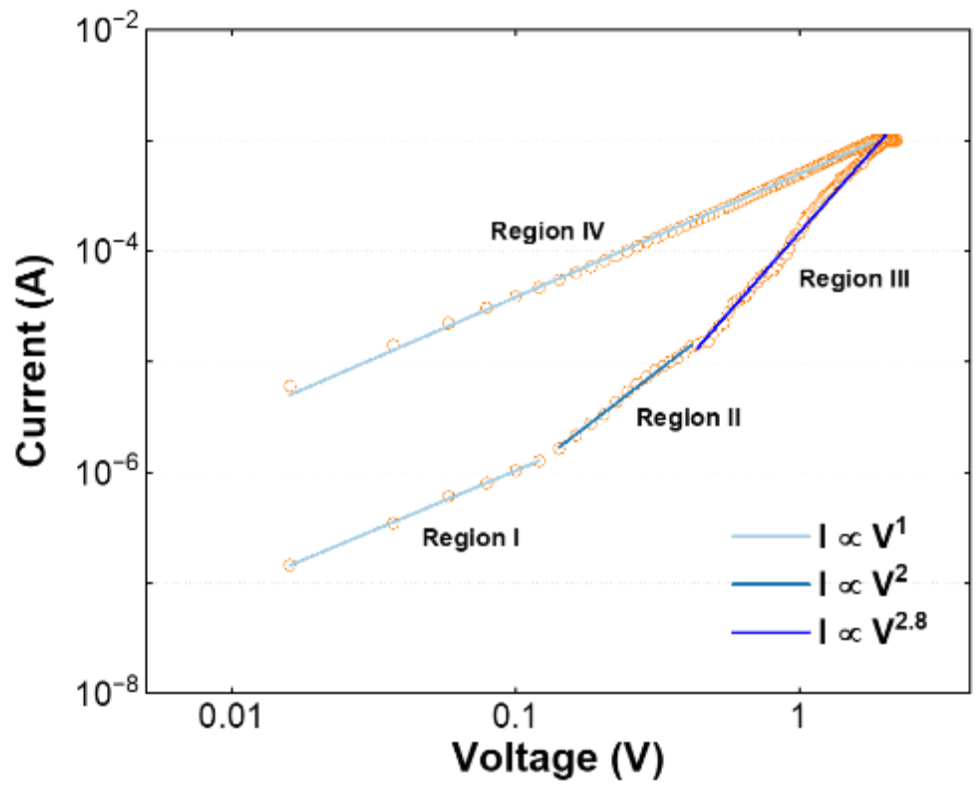

Figure S12. Log–log I–V characteristics of the nanoporous SiO_2 memristor in nonvolatile switching mode, illustrating the evolution of conduction from initial ohmic behavior to trap-controlled SCLC, and finally to filamentary ohmic transport in the low-resistance state.

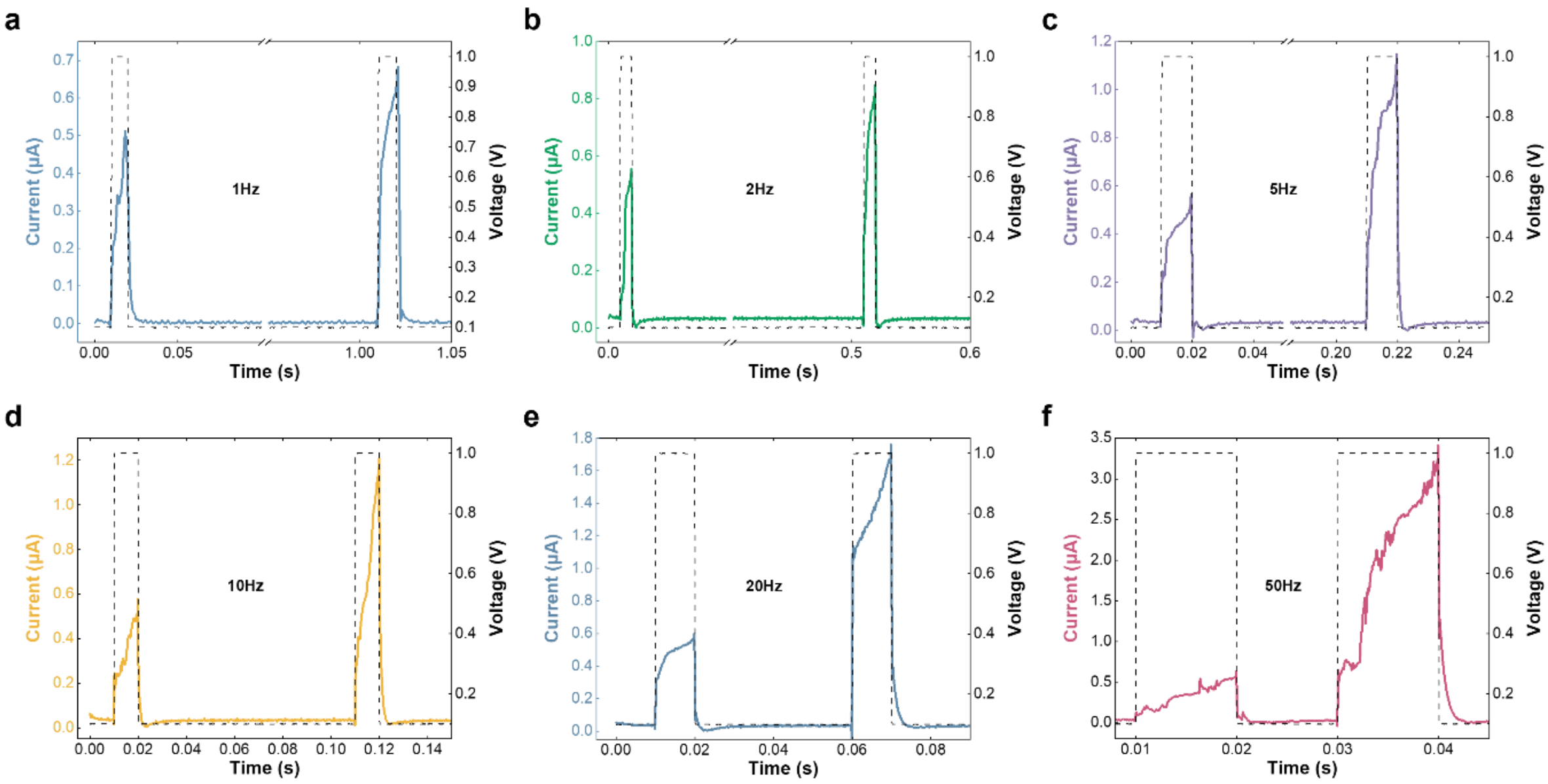

Figure S13. In-pulse PPF measurements at the indicated frequencies (1, 2, 5, 10, 20, and 50 Hz).

Table S2. Extracted LTP/LTD fitting parameters over five repeated update cycles based on the exponential conductance-update model. Dynamic range (G_{max}/G_{min}) represents the conductance modulation window, β quantifies the nonlinearity of conductance modulation, and R^2 indicates the fitting quality. The relatively small variations in fitting parameters together with high R^2 values indicate acceptable cycle-to-cycle reproducibility. These results further demonstrate stable analog weight-update behavior during repeated pulse training.

Cycle	G_{min} (μS)	G_{max} (μS)	Dynamic range	β_P	R_P^2	β_D	R_D^2
1	59.69	292.44	4.90	2.18	0.990	3.63	0.994
2	73.50	332.84	4.53	2.84	0.869	4.48	0.937
3	71.39	321.18	4.50	3.05	0.947	4.66	0.940
4	78.02	309.16	3.96	3.77	0.982	4.04	0.969
5	79.11	313.14	3.96	3.62	0.981	4.18	0.965
Mean	72.34	313.75	4.37	3.09	0.954	4.20	0.961
SD	7.75	14.96	0.41	0.64	0.05	0.40	0.023

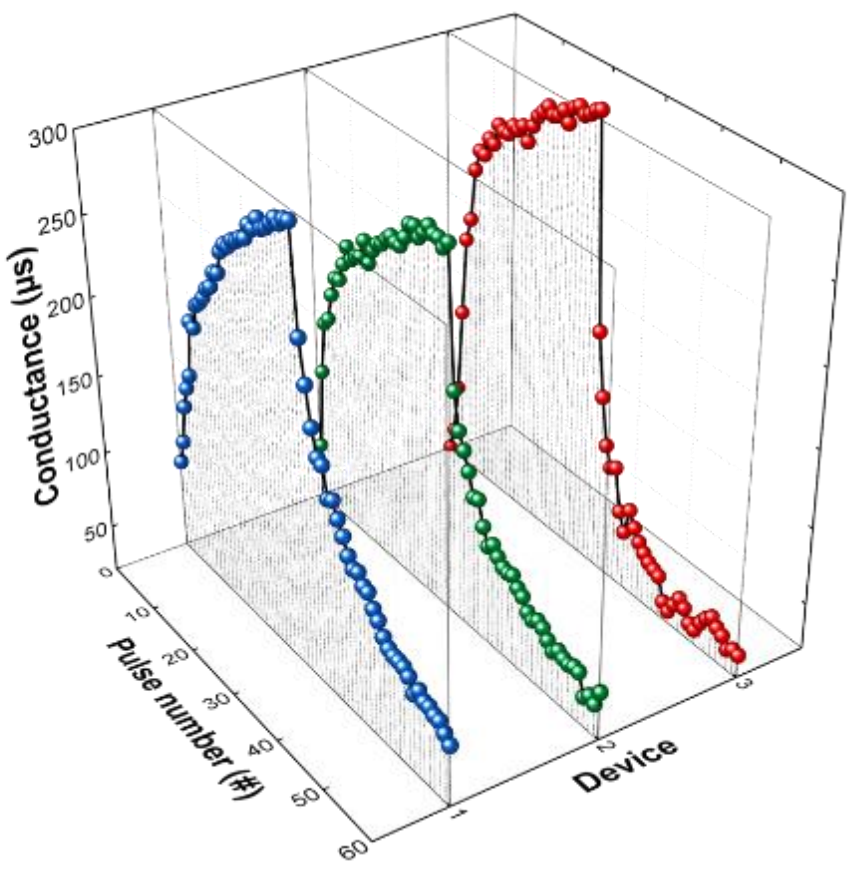

Figure S14. LTP/LTD characteristics measured from three different devices, confirming good device-to-device consistency in analog switching behavior.

Table S3. Cumulative probability of state retention after positive-pulse programming

Pulse Number (#)	σ/μ (%)	Pulse Number (#)	σ/μ (%)
**1+**	2.078	**15+**	0.029
**2+**	2.078	**17+**	0.222
**3+**	2.967	**19+**	0.029
**4+**	1.324	**21+**	0.029
**5+**	1.483	**23+**	0.025
**7+**	0.101	**25+**	0.330
**9+**	0.174	**27+**	0.278
**11+**	0.775	**29+**	0.315
**13+**	0.536	**30+**	0.148

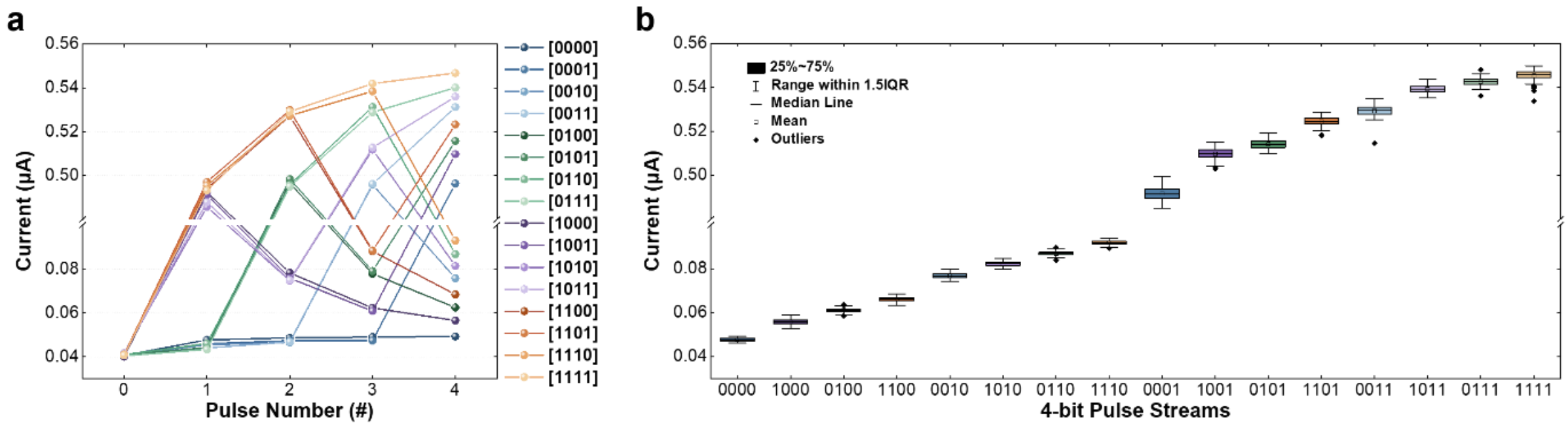

Figure S15. Reservoir state evolution and cycle-to-cycle reliability of the nanoporous SiO_2 memristor. (a) Evolution of current values of 16 reservoir states for [0000]-[1111] pulse inputs, showing distinct and well-separated transient trajectories. (b) 100 repeated cycles for each 4-bit state, demonstrating minimal drift and excellent reproducibility across all states.

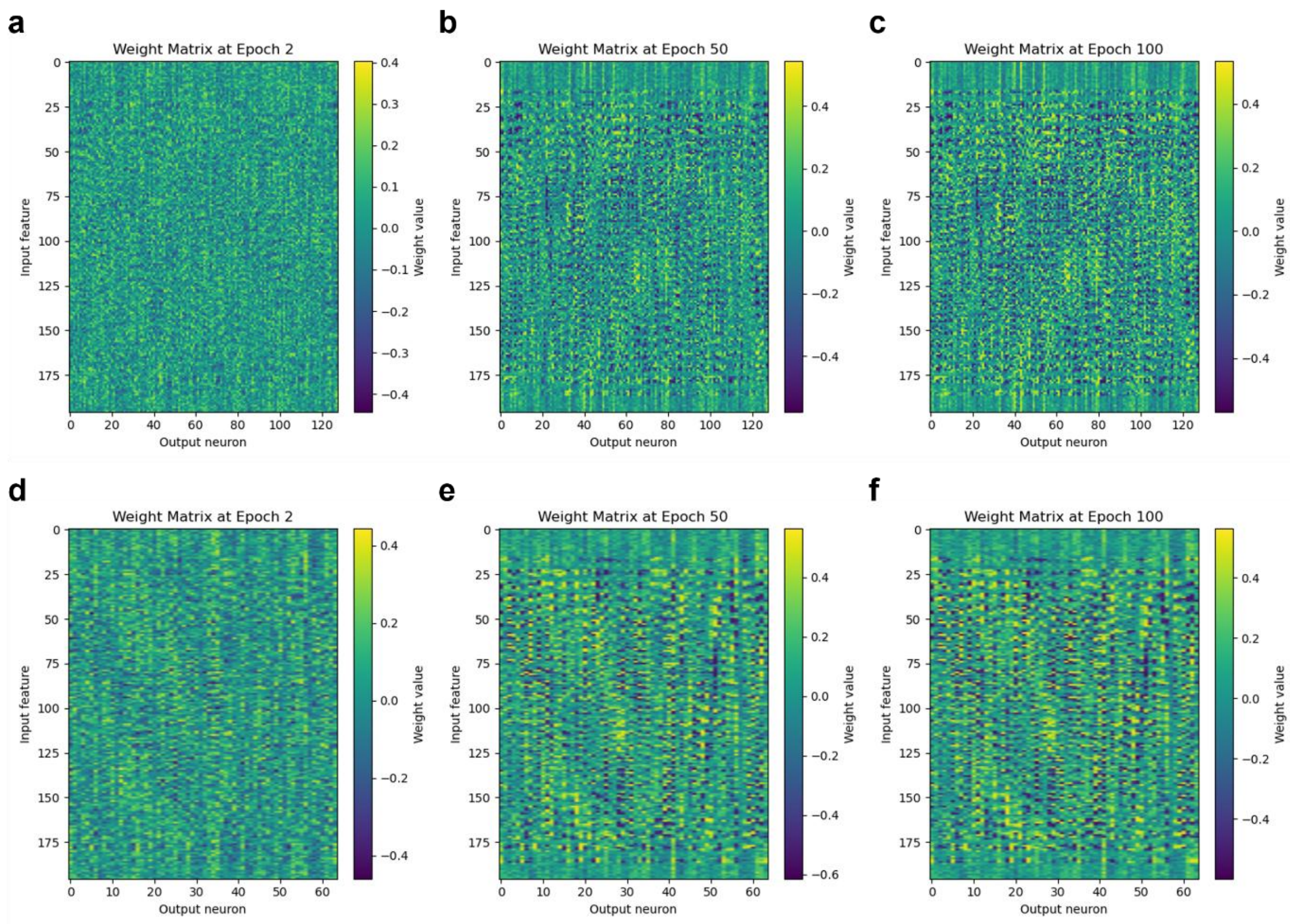

Figure S16. Evolution of hidden-layer weights during MNIST handwritten-digit recognition: (a-c) Under the mean-fitting condition, heatmaps of the hidden-layer weight matrix at epochs 2, 50, and 100, respectively. Each panel shows the normalized weight magnitudes; brighter regions indicate larger weights. (d-f) Under the variability-aware condition, heatmaps of the hidden-layer weight matrix at epochs 2, 50, and 100, respectively. Each panel shows the normalized weight magnitudes; brighter regions indicate larger weights.

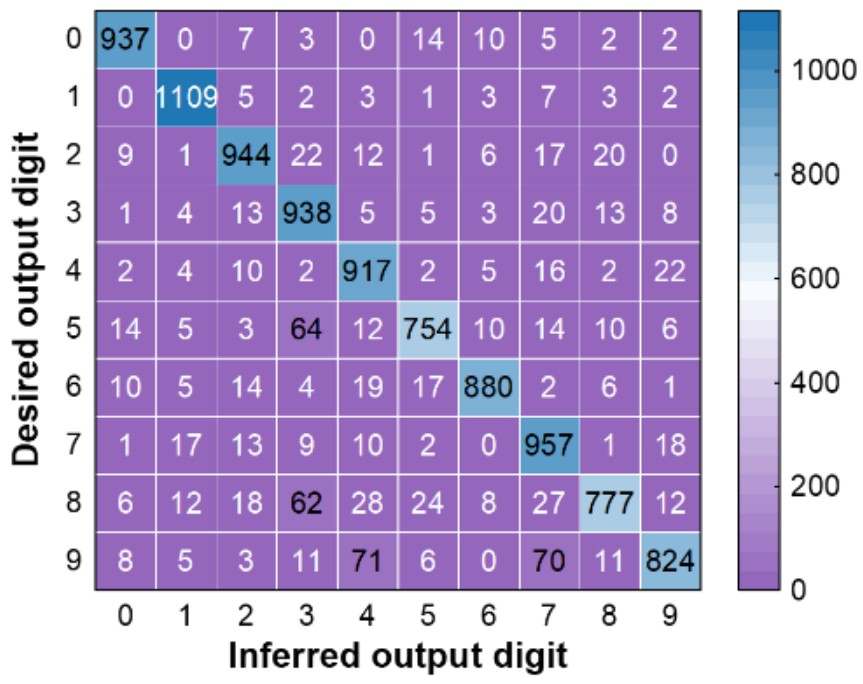

Figure S17. Confusion matrix obtained under the variability-aware condition for 10,000 MNIST test images, confirming accurate recognition across all digit categories.

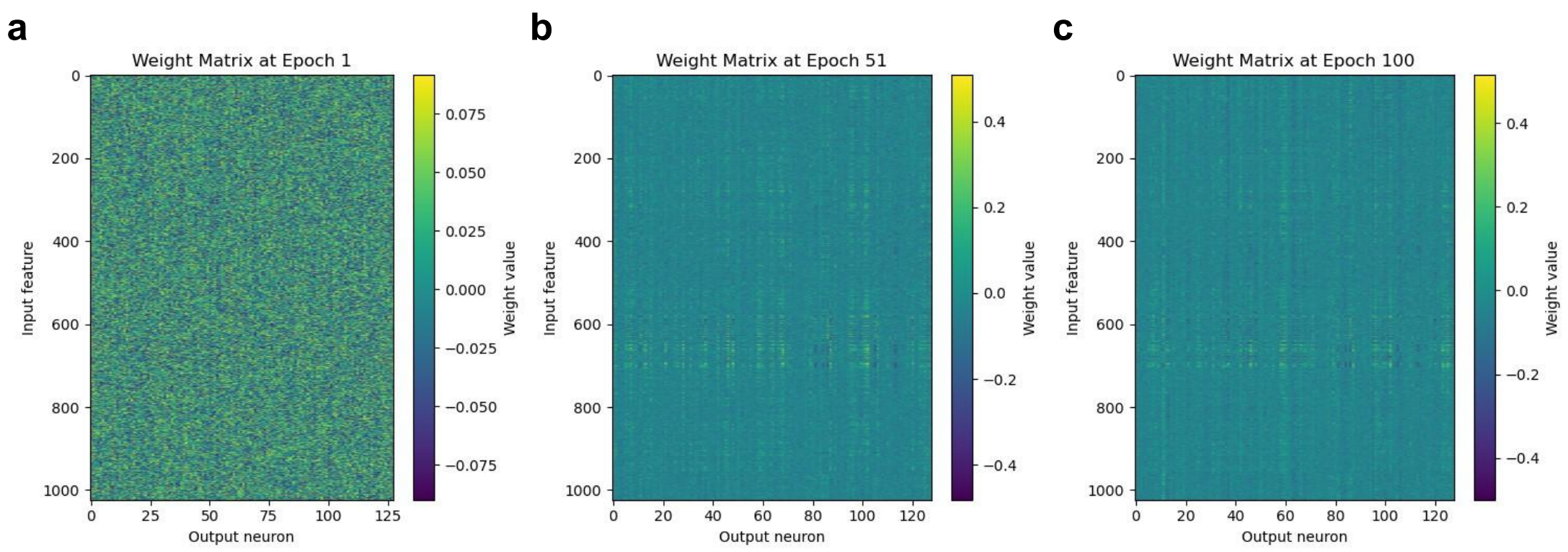

Figure S18. Evolution of hidden-layer weights during ECG biosignal recognition: (a-c) Heatmaps of the hidden-layer weight matrix at epochs 1, 51, and 100, respectively. Each panel shows the normalized weight magnitudes; brighter regions indicate larger weights.

References

[1] S. Munjal, N. Khare, *Nanotechnology* **2021**, *32*, 185204.
[2] E. Passerini, M. Lewerenz, M. Csontos, N. Jimenez Olalla, K. Keller, J. Aeschlimann, F. Xie, A. Emboras, X. Zhang, M. Fischer, Y. Fedoryshyn, M. Luisier, T. Schimmel, U. Koch, J. Leuthold, *ACS Appl. Electron. Mater.* **2023**, *5*, 6842.
[3] K. S. Woo, H. Park, N. Ghenzi, A. A. Talin, T. Jeong, J.-H. Choi, S. Oh, Y. H. Jang, J. Han, R. S. Williams, S. Kumar, C. S. Hwang, *ACS Nano* **2024**, *18*, 17007.
[4] M. Ismail, E. Seo, M. Rasheed, Y. Park, C. Mahata, S. Kim, *J. Chem. Phys.* **2024**, *161*, 224701.
[5] I. Oh, J. J. Pak, *J. Alloys Compd* **2025**, *1020*, 179383.
[6] R. Islam, Y. Shi, G. V. de Oliveira Silva, M. Sachdev, G.-X. Miao, *ACS Nano* **2024**, *18*, 22045.
